# Protein Quality in Perspective: A Review of Protein Quality Metrics and Their Applications

**DOI:** 10.3390/nu14050947

**Published:** 2022-02-23

**Authors:** Shiksha Adhikari, Marijke Schop, Imke J. M. de Boer, Thom Huppertz

**Affiliations:** 1 Food Quality & Design Group, Wageningen University & Research, 6708 WG Wageningen, The Netherlands; shiksha.adhikari@wur.nl; 2 Animal Production Systems Group, Wageningen University & Research, 6708 WD Wageningen, The Netherlands; marijke.schop@wur.nl (M.S.); imke.deboer@wur.nl (I.J.M.d.B.); 3 Friesland Campina, Research and Development, 3818 LE Amersfoort, The Netherlands

**Keywords:** protein quality, indispensable amino acids, digestibility, food processing, complementarity

## Abstract

For design of healthy and sustainable diets and food systems, it is important to consider not only the quantity but also the quality of nutrients. This is particularly important for proteins, given the large variability in amino acid composition and digestibility between dietary proteins. This article reviews measurements and metrics in relation to protein quality, but also their application. Protein quality methods based on concentrations and digestibility of individual amino acids are preferred, because they do not only allow ranking of proteins, but also assessment of complementarity of protein sources, although this should be considered only at a meal level and not a diet level. Measurements based on ileal digestibility are preferred over those on faecal digestibility to overcome the risk of overestimation of protein quality. Integration of protein quality on a dietary level should also be done based on measurements on an individual amino acid basis. Effects of processing, which is applied to all foods, should be considered as it can also affect protein quality through effects on digestibility and amino acid modification. Overall, protein quality data are crucial for integration into healthy and sustainable diets, but care is needed in data selection, interpretation and integration.

## 1. Introduction

Food and nutrition play a crucial role in the maintenance of human health and the prevention of non-communicable diseases. It has been estimated that in 2017, 11 million deaths and 255 million disability-adjusted life years (DALYs) were attributable to dietary risk factors [1]. The main risk factors identified are diets high in sodium, diets low in whole grains, diets low in fruit, diets low in vegetables and diets low in nuts and seeds. Other factors include diets low in milk, diets high in sugar-sweetened beverages and diets high in processed meat [1]. Management of these dietary risk factors requires a balanced composition of the human diet. Composing an optimal diet from a nutritional perspective should focus on ensuring that all essential nutrients are provided at the required levels, while at the same time also on ensuring that this is done through a combination of food products which does not result in excess intake of nutrient or non-nutrient components which can lead to dietary risk factors [2]. In other words, ensuring sufficient intake of an essential nutrient like vitamin C should not be in the form of sugar-sweetened beverages considering that diets high in these products increase risks of cardiovascular diseases and type 2 diabetes. 

To assist the public, but also policy-makers and health professionals, in improving eating patterns and select healthy diets, dietary guidelines are published in many countries. These dietary guidelines are often based on food groups, e.g., fruits and vegetables, dairy, meat, fish, grains, etc. and recommend a minimum or maximum intake of food products from each group [3,4]. In the past two decades, however, it has become abundantly clear that diets good for human health are not necessarily good for planetary health and that in addition to human nutrition, the impact of food production on planetary health should also be considered [5,6]. Based hereon, the EAT-Lancet commission on healthy diets from sustainable food systems introduces a global planetary health diet that was designed to be optimal for both human health and planetary health [7]. This proposed diet called from a shift from animal-based to plant-based foods [7], a recommendation voiced by others as well, primarily based on the environmental impact of animal-based food production [5,8,9,10,11].

However, it has also been argued extensively that exclusively plant-based diets entail risks of nutrient deficiencies because some essential nutrients are not present, or only present at low amounts, in plant-based foods, e.g., vitamin B12 or iodine [12,13,14,15]. For other essential nutrients, e.g., calcium (Ca) or zinc (Zn), plant-based foods may be able to supply reasonable levels of intake, but low bioavailability of these minerals in many plant-based foods, due to the presence of phytates or oxalates, leads to risks of deficiencies [15,16,17,18,19]. In addition to micronutrients, concerns have also been raised over one of the macronutrients in relation shifts to more plant-based diets, i.e., protein [8,20,21]. The class of dietary proteins is extremely diverse, with notable variations both in amino acid composition and in digestibility of the protein between different sources of dietary protein. As both the right amino acid composition and high digestibility are required for proteins to meet the requirements of the human body, the ability of dietary proteins to meet these requirements varies widely [22,23]. Such abilities are often quantitatively expressed in so-called protein quality metrics, which include amino acid composition and digestibility [22,23]. It has been argued that such protein quality metrics should be considered in the assessment of environmental impact of food proteins. Recent publications in this field have highlighted that the consideration of protein quality notably affects consideration of the environmental impact of dietary proteins [24,25]. 

While consideration of protein quality in the dietary context and in view of dietary shifts aimed at both human and planetary health is thus important, the implementation of this concept is not easy. Reported protein quality metrics can be used to rank proteins, the inclusion of such metrics in a dietary perspective is more complex because protein, unlikely, e.g., vitamin C or Ca, is not a single nutrient, but the carrier of many essential nutrients, i.e., 9 indispensable amino acids, plus dietary nitrogen. Integration of protein quality in a dietary perspective thus requires consideration of multiple aspects. In this paper, we review the concept of protein quality, with particular emphasis on the inclusion of this concept in dietary considerations. For this purpose, the importance of protein for human nutrition and health, as well as protein digestion and amino acid absorption are covered in Section 2. Subsequently, Section 3 covers the different methodologies that have been applied to determine protein quality. Section 4 considers the interpretation and application of data derived from protein quality measurements. Finally, Section 5 and Section 6 cover two important aspects for consideration of protein quality on a dietary basis. The effect of processing and preparation of food products is the focus of Section 5. Section 6 covers the complementarity of different proteins on a dietary basis, but with that also outlines the crucial importance of the time scales at which complementarity is considered, i.e., on a meal level rather than on a diet level. Finally, in Section 7, we provide some conclusions and perspectives on future steps for including the concept of protein quality in dietary recommendations. 

## 2. Protein Quality

### 2.1. The Importance of Proteins and Amino Acids for Human Nutrition and Health

Next to carbohydrate and fat, protein is one of the macronutrients. Digestible carbohydrates are only a source of energy to the human body, whereas dietary fat is a source of energy as well as a source of the essential fatty acids linoleic acid (LA) and α-linolenic acid (ALA), which cannot be synthesized by the human body. Adequate intake (AI) levels for LA and ALA for adults were defined by EFSA as 4% and 0.5% of total energy intake (EI), whereas total fat intake was recommended to be between 20% and 35% of EI [26]. Hence, while dietary fat is not only a source energy for the human body, 78–87% of fat is actually used as a source of energy at the recommended intake levels. Like carbohydrates and fat, protein can also be a source of energy. However, far more importantly, proteins are the main dietary source of nitrogen and indispensable amino acids (IAAs), which are required by the body for protein synthesis to enable e.g., tissue growth and maintenance [27].

Proteins play a crucial role in the growth, maintenance and physiological functions of the human body [28]. All amino acids are important in the synthesis and functioning of muscles and organs, as well as in enzymes, hormones and the immune system [29]. Amino acids are classified as dispensable amino acids (DAAs) and IAAs, based on whether or not the body can synthesize the particular amino acid. DAAs can be synthesized *de novo* by the human body [29], whereas IAAs cannot by synthesized by the human body and the only source of IAAs is dietary protein; hence, it is important to assure adequate dietary intake of IAAs [30]. In addition, some DAAs, such as arginine, cysteine, glutamine, glycine, proline and tyrosine, can become conditionally indispensable, e.g., for premature neonates [27]. In these cases, the body cannot produce sufficient levels of these amino acids and these amino acids thus become conditionally IAAs, and need to be supplied through dietary protein to compensate for insufficient synthesis in these stages of life [27,31,32]. To meet the metabolic demand and to assure proper functioning of the human body, consumption of adequate amounts of protein is thus essential to meet both total nitrogen and IAA requirements [33].

The general dietary requirement for protein is defined as an estimated average requirement (EAR) and recommended dietary allowance (RDA). The EAR is the daily intake level for a nutrient that is estimated to meet the requirement for 50% of the target population, whereas the RDA, which is calculated as the EAR plus two times the standard deviation, meets the requirements for 97–98% of the population [34]. For all adults above 18 years of age, the EAR for protein is 0.66 g protein per kg body weight per day and the RDA is 0.83 g protein per kg body weight per day [27]. EAR and RDA values for children less than 18 years and for pregnant and lactating women are higher than for the general adult population [27]. Studies have also suggested that protein requirements for elderly adults could be higher, as summarized by [27], and that amino acid requirements can be amended to minimize aging-related health outcomes [35], but these findings have not yet been translated into clear recommendations by authorities [27]. Further details on protein requirements throughout life cycle are described in further detail elsewhere [36]. No upper limits for protein intake, or the intake of specific amino acids, have been defined to date. However, findings in the novel area of dietary protein restriction, as recently reviewed [35], warrant further consideration in future. 

In addition to total protein intake, requirements for each IAA have also been defined [30,32]. The requirements for IAAs as defined by FAO [30,32] and EFSA [27] are presented in Table 1. Similar to RDA values for total protein [27], IAA requirements are highest for the 0.5–1-year-olds, and decrease progressively with increasing age (Table 1). The decreasing requirements for each IAA with increasing age reflect the fact that in the early stages of life, the IAAs are required for growth, development and maintenance of the body, whereas in later stages of life, requirements for growth and development progressively decrease and requirements for IAAs are mainly based on maintenance [37,38]. Considering the requirements for IAAs (Table 1) and the fact that protein is the only dietary source of IAAs, an RDA for protein thus does not only contain a quantitative aspect but also a qualitative aspect; i.e., the RDA of 0.83 g protein/kg bodyweight/d for adults is only sufficient to meet the requirements for target population if this intake also provides the levels of IAAs outlined in the Table 1. For a protein that cannot meet these IAA levels at the RDA for protein intake, either intake at the RDA level can lead to insufficient intake of one or more IAAs or higher intake levels than the RDA are needed to achieve recommended intake of IAA. Such aspects are central to the concept of protein quality.

### 2.2. Protein Digestion and Absorption by Humans

**Figure 1:**
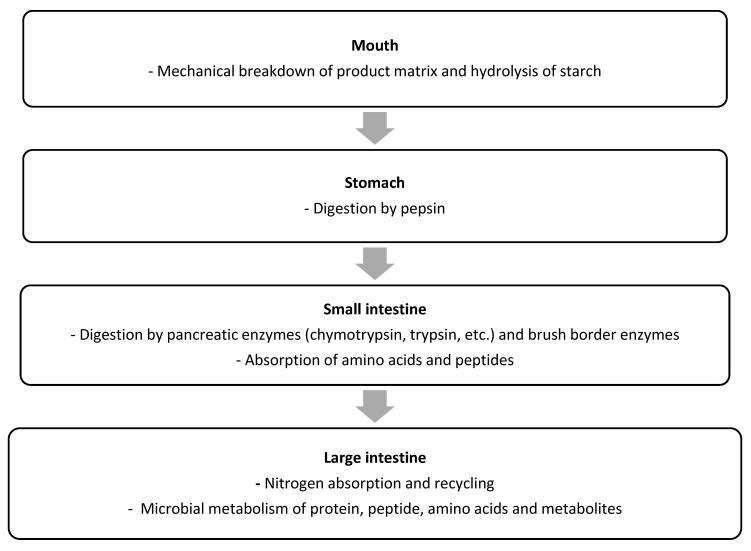
Schematic overview of the key steps of protein digestion and absorption in humans.

As outlined earlier, proteins are the key source of IAAs for the human body. However, for the IAAs, as well as the DAAs, from a protein to be utilized by the human body, the protein first needs to be hydrolysed into free amino acids and small (di- and tri-) peptides, which can subsequently be absorbed in the bloodstream [39]. Protein digestion is a complex, multistage process, as schematically outlined in Figure 1. The protein digestion process begins with the mechanical disruption of the product matrix containing the protein during mastication in the mouth. While protein breakdown does not occur at this stage, the oral phase of digestion can be important for protein digestion, because by disrupting the product matrix, the surface area increases, which increases exposure of the protein to digestive proteases and peptidases during the later stages of digestion [40,41]. Digestion of starch by salivary amylase can also disrupt the product structure, thereby increasing accessibility of protein to digestive enzymes in later stages of the digestion process [41].

Following the oral phase of digestion, which typically lasts only for a short time (<1 min), the product passes on to the stomach, where the gastric phase of the digestion process takes place. In this phase, the product is mixed with gastric juice, which has a low pH (1–2 for adults) and contains the protease pepsin [42,43]. The mixing of gastric juice and product is facilitated through contractions of the stomach. In the stomach, some hydrolysis of proteins by pepsin occurs, resulting in (poly)peptide formation [42], but complete protein digestion to free amino acids and peptides small enough for absorption does not occur at this stage [44].

Following gastric digestion, the chyme is delivered to the duodenum at a rate determined by the gastric emptying. In the small intestine, the chyme is mixed with pancreatic proteases and peptidases, such as trypsin, chymotrypsin, and carboxypeptidase A [40]. Together with intestinal brush border enzymes, these enzymes hydrolyse the proteins and (poly)peptides into amino acids, di-, tri-, and oligopeptides [45,46]. Pancreatic proteases and peptidases are considered rigorous compared to pepsin, and most of the protein digestion occurs in the small intestine rather than in the stomach [46]. The amino acids and di- and tripeptides that are released can be taken up across the small intestinal mucosa and are generally considered to be almost fully absorbed by the end of small intestine, i.e., the terminal ileum [45]. The amino acids and peptides not absorbed at the terminal ileum pass to the large intestine. The large intestine, especially the caecum, also contains amino acid transporters, but there is thus far no evidence that the absorption of amino acids in large intestine occurs in relevant quantities [47,48]. If fully absorbed, the amino acids absorbed in the large intestine in pigs would increase the level of total amino acids absorbed by only 0.1% for whey protein and by 3.5% for zein [47]. The quantity of amino acids passing to large intestine is thus only affected by the absorption of amino acids and small peptides from the consumed protein to a limited amount. The proportion of unabsorbed amino acids and peptides, as well as of undigested protein and polypeptides, can also be digested and fermented by the microbiota [48,49]. Furthermore, colonocytes are capable of synthesizing and metabolizing amino acids which are likely derived through blood circulation rather than the digesta [48]. Given that protein is mainly digested and absorbed in the small intestine, and microbial protein is formed in the large intestine, digesta samples collected from either site may differ notably, and cause variation in measurements of protein digestibility [50]. This is an important factor in the consideration of the different methods used for determining protein quality, which are described in Section 3.

## 3. Protein Quality Measurement

### 3.1. Defining Protein Quality

**Figure 2:**
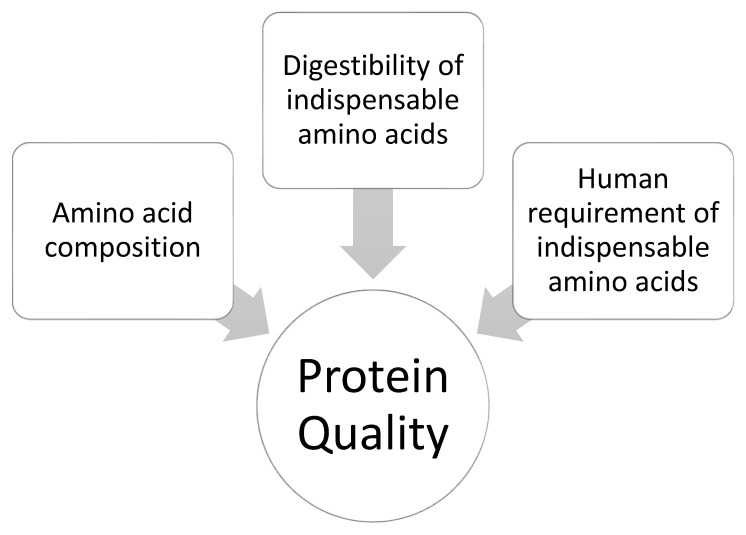
Elements required to quantitively define protein quality.

As outlined in Section 2, with the existence of RDA values for total protein and requirement values for IAAs, protein requirements include both a quantitative and qualitative aspect. The adequacy of a dietary protein to meet the IAA requirements of humans is often considered the basis of expression of protein quality. Several commonly used principles of expressing protein quality are based on the ability of a protein source to supply sufficient IAA for a specific target group [51,52], and hence encompasses the three essential elements outlined in Figure 2: (i) amino acid composition, (ii) digestibility of the IAAs and (iii) IAA requirements of the target population [53], whereby amino acid composition and requirements for each IAA are typically expressed per g of protein. The requirements for the IAAs are combined in a so-called reference protein that, based on 100% digestibility, contains all IAA at the required level per gram of protein. This reference protein, or scoring pattern, is developed for different age groups [54], e.g., for different age categories, as shown in Table 2 [27,30]. Digestibility of protein is typically defined as the proportion of ingested protein that is hydrolysed into amino acids, di- and tripeptides, which are available for absorption [55]. The concentration and digestibility of the IAAs in a protein thus determine the overall protein quality [30], and large variations in protein quality are observed among food [30,56]. Some dietary proteins contain all IAAs in digestible form at levels that are adequate to meet the requirements in Table 1, whereas in other dietary proteins, one or more IAAs may not be present at the required level in digestible form [57]. 

**Figure 3:**
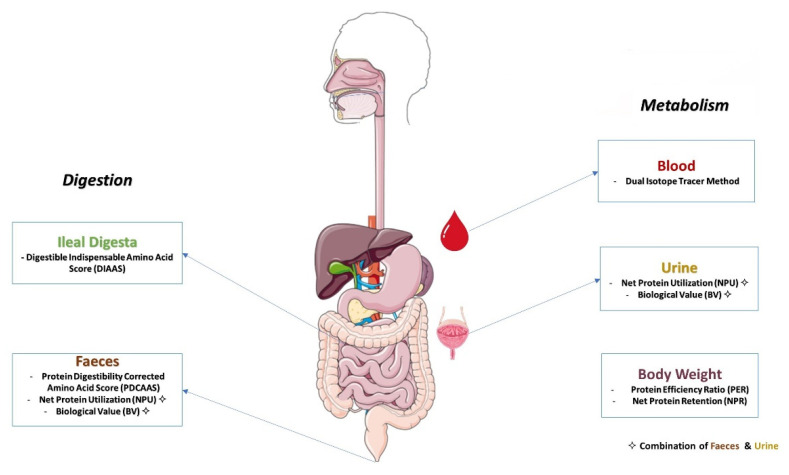
Overview of site of measurement for different in vivo protein quality measurement methods.

Over the years, many different methods have been developed and implemented for the determination of protein quality. These methods follow different principles to quantify or classify protein quality of a protein source. The different principles used in these methods are outlined in Table 3, which includes the parameters measured and equations used to calculate the quality of protein. A schematic outline of the different parts of the human body sampling for measurements for each method is shown in Figure 3. The different methods used for determining protein quality are covered in detail in Section 3.2 and Section 3.3. These methods vary in several aspects; i.e., they may be in vivo or in vitro and for in vivo methods, they may be carried out in humans or in animals. Furthermore in vivo trials in humans or animals may differ in the point of sampling, with main differences being sampling for digesta at the terminal ileum or in the faeces, which have a major impact on the outcomes of protein quality measurements. Section 3.2 will focus on protein quality methods that are either fully or partially based on the elements outlined in Figure 2 [30]. In addition to protein quality methods based on amino acid digestibility, there are also several methods based on growth studies, whereby growth of animals (typically determined as weight gain) is determined for a diet containing a test protein and compared to a reference protein. These are described in Section 3.3. Furthermore, in vitro method for protein digestion and protein quality have been described, which are briefly covered in Section 3.4. 

### 3.2. Methods for Determining Protein Quality Based on Amino Acid Digestibility

From Table 3 and Figure 3, it clear that there is a wide variety of methods available for determining protein quality. Considering, however, that, as outlined in Section 3.1, the expression of protein quality should reflect the adequacy of a protein to meet the IAA requirements of humans, protein quality should be ideally measured in vivo on an amino acid basis, whereby digestibility is considered and the amount of digestible IAAs is compared to a reference pattern of IAA requirements [54]. This allows placing protein quality in a broader context, i.e., not only considering it as a metric for comparing or ranking individual protein sources, but also in a broader dietary perspective, where multiple protein sources are included and complementarity among protein sources is also an important consideration, which is further discussed in Section 6. These protein quality measurements required the use of digesta, which may be ileal or faecal and may be from humans or animals. 

**Figure 4:**
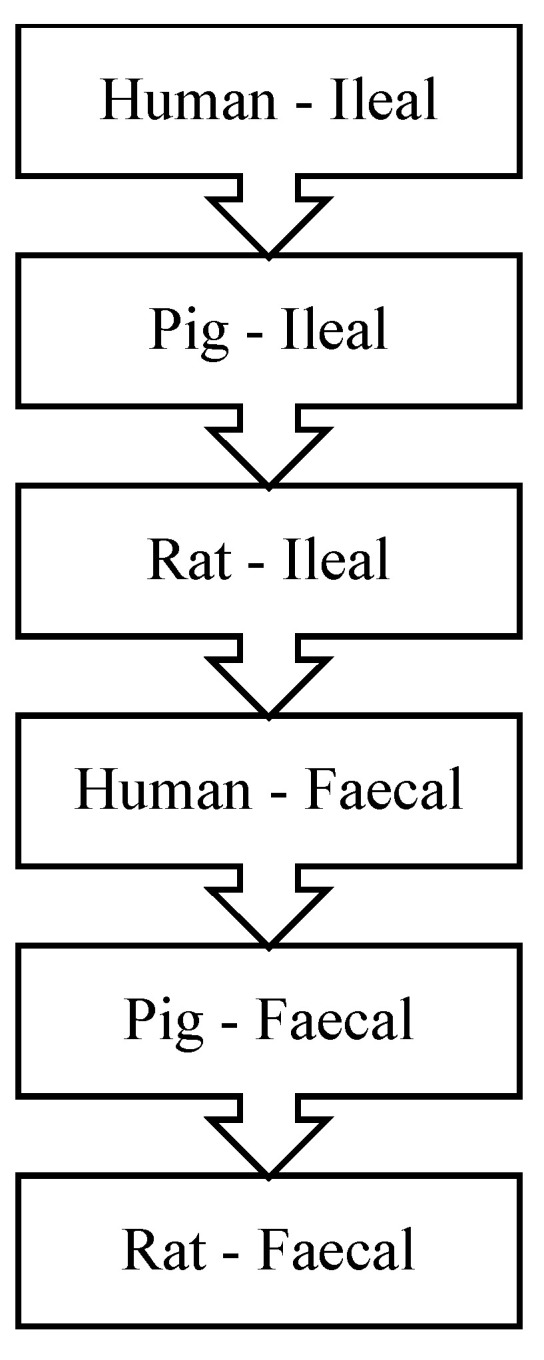
Ranking preference of digesta used for determination of protein quality. Redrawn from [63].

The preference ranking of the different digesta based on the representativeness to human digestibility for protein quality measurements as suggested by FAO [63] is presented in Figure 4. This ranking indicates that ileal digesta are preferred over faecal digesta and that humans are preferred over pigs, which are preferred over rats. Overall, ileal digesta from humans are thus most preferred for assessment of protein quality. Some studies have indeed been conducted in humans to determine the ileal digestibility of proteins [64,65,66]. However, ileal digesta collection in human is complicated [67], and most data available to date on ileal digestibility of dietary proteins has come from animal models [68]. Based on Figure 4, ileal digesta from pigs and rats are preferred after human ileal digesta, but over human faecal digesta [63]. The endorsed use of pigs and rats as animal models for studying protein digestibility is based on their physiological resemblance and close correlation of protein digestibility with that of humans [69]. Overall, pigs are preferred over rats because the anatomy and physiology of digestive tract of growing pigs is more similar to that of adult humans [22,69]. Indeed, true ileal digestibility of dietary protein in humans and pigs have shown a high degree of correlation across different foods [66,69]. Studies in rats have also shown a good general agreement to humans for true ileal amino acid digestibility, although it has been reported that rats can potentially better digest some proteins compared with humans [69]. These findings make the available data on pig and rat models valuable to calculate protein quality in relation to human requirements. In the absence of ileal digestibility data, faecal digestibility can be considered (Figure 4). However, faecal digestibility of total nitrogen is acceptable to be considered, it is not the case when it comes to AA digestibility [70,71].

Of the different methods for protein quality measurement outlined in Table 3, two methods stand out for the purpose of determining protein quality at an amino acid level, i.e., the digestible indispensable amino acid score (DIAAS) method [30] and the protein digestibility-corrected amino acid score (PDCAAS) method [51]. The DIAAS method calculates a score for each IAA based on the concentration of each digestible IAA, calculated from the concentration of each IAA (per g protein) and its ileal digestibility [30,50,60]. For each IAA, this value is then compared with a refence pattern for each age group (see Table 2) and the IAA with lowest score relative to the reference pattern is considered to be the first limiting IAA (IAA_lim_); the DIAAS value for the protein is taken as the score for the IAA_lim_ [60]. For calculation of the DIAAS value, FAO recommends using scoring patterns for 0–6-month-old babies based on the amino acid composition of human milk, whereas scoring patterns for 0.5–3-year-old children are based on values for 0.5–1-year-old category and values for the older child (>3 years old), adolescents and adults are based on the 3–10-year-old age group (Table 2; [30]). The DIAAS method has been extensively used in the past decade to study protein quality in food products and food protein ingredients. Outcomes of these studies are discussed in Section 4. 

Due to its longer history of use, the PDCAAS method has been applied more broadly than the DIAAS method [28]. Like the DIAAS method, the PDCAAS method calculates a value for protein quality based on the first limiting amino acid in relation to a reference pattern [59]. However, there are notable differences between the DIAAS method and the PDCAAS method [72]:

- digestibility in the PDCAAS method is not determined at the ileal but at the faecal level in test species, and
- digestibility in the PDCAAS method is determined at a protein level, and not at the individual amino acid level, and the protein digestibility factor is subsequently applied to every individual IAA.

Like for the DIAAS method, the calculated digestible amino acid level in the PDCAAS method is subsequently compared to the reference pattern and the score for the first limiting amino acid is indicated as the PDCAAS value for the protein, with values truncated at 1 or at 100% [30]. With data available for a large range of foods and the comparative ease of measurement, the PDCAAS method has been widely used in the past, and is still being used, to determine protein quality of food products [28]. However, the PDCAAS method has come under criticism for several aspects:

- the determination of faecal rather than ileal digestibility in the PDCAAS method, despite the fact that it is established that amino acids absorbed past the terminal ileum do not contribute to protein metabolism and that faecal nitrogen levels may be affected by nitrogen metabolism of gut microbiota [28];
- the fact that digestibility in the PDCAAS method is determined on a protein basis, rather than on an individual amino acid basis, despite the fact that it is known that digestibility values between amino acids in protein sources vary widely [30];
- the truncation of protein quality scores at 100% in the PDCAAS method not allowing to consider complementarity of different protein sources on an amino acid basis (for further explanation see Section 6).

**Figure 5:**
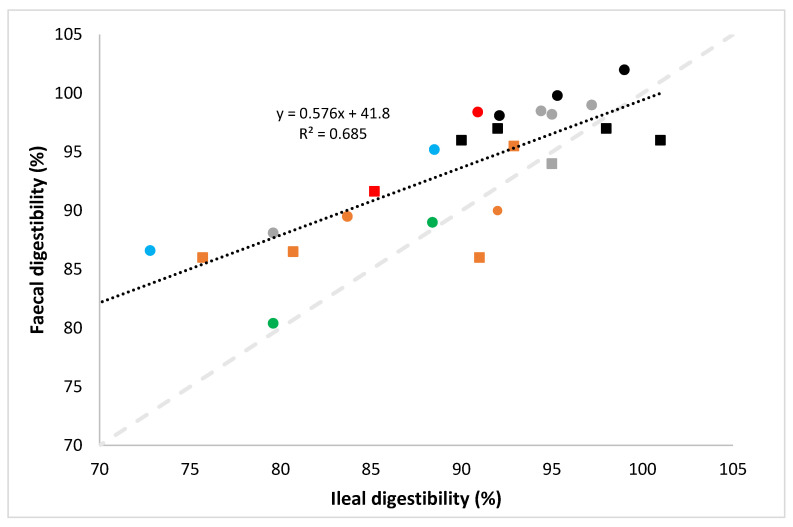
Relation between faecal digestibility and ileal digestibility protein from milk and milk protein concentrates and isolates (●, ■), roasted nuts (●, ■), cooked cereals (●), raw cereals (●, ■), legume and cereal protein isolates and concentrates (●, ■) and cooked legumes (●) tested in pigs (squares) or rats (circles). Data from [57,72,73,74]; Black dotted line: trend line based on linear regression; grey dashed line: line of unity.

It has been indicated that the PDCAAS method can overestimate the protein quality, especially of protein sources with poor digestibility, due to the use of faecal rather than ileal digestibility measurements [57,72,73]. To illustrate this, data were collected from studies that directly compared ileal and faecal digestibility of dietary proteins in either pigs or rats. From the results shown in Figure 5, it is clear that for most products faecal protein digestibility was higher than ileal digestibility. In some cases, the overestimation of faecal digestibility compared to ileal digestibility exceeds 10% (Figure 5). Considering that, as outlined earlier, any amino acids absorbed post the terminal ileum are unlikely to contribute to the metabolic amino acid pool [47], measurement of faecal digestibility, as used in the PDCAAS method, results in the risk of overestimating protein quality, compared to measurements based on ileal digestibility used in the DIAAS method. One study in pigs also reported a lower digestibility score for faecal digestibility compared to ileal digestibility, which may be due to nitrogen secretion into hindgut [57]. In case of dietary protein with low protein digestibility, a lower degree of correlation between the ileal and faecal digestibility has been reported previously [71], which was also observed in Figure 5. 

**Figure 6:**
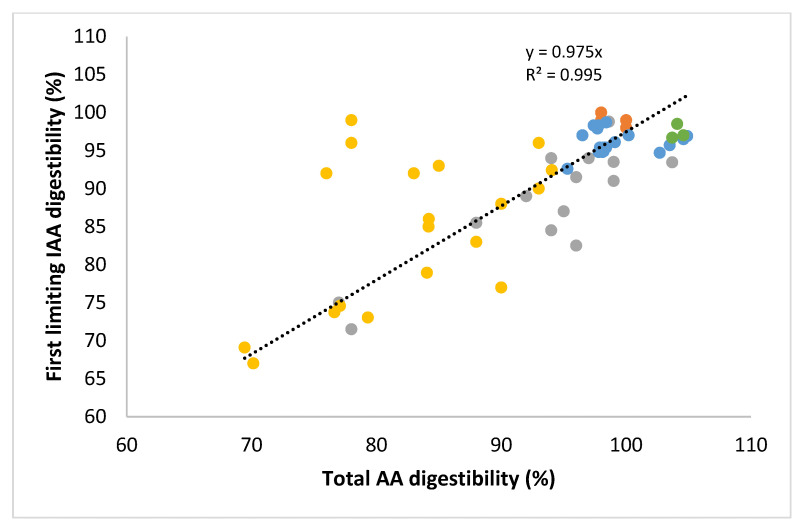
Relation between ileal digestibility of total amino acids (AA) and of the first limiting indispensable amino acid (IAA) histidine (●), sulphur-containing amino acids (●), lysine (●), valine (●) or leucine (●) of different food sources. Data from studies in pigs [57,76,77,78,79,80], and rats [72,81].

In addition to differences in the type of digesta being analyses (i.e., ileal or faecal), differences in outcomes between the DIAAS method and the PDCAAS method for protein quality measurement are suggested to be due also to the fact that the former determines the digestibility of each IAA individually, whereas the latter determines protein digestibility and applies this factor to each IAA [60]. Because of differences in the digestibility between different IAAs [70,75], it has been suggested that only determining protein digestibility impacts outcomes [50]. To explore this, we studied the relationship between standardized ileal digestibility (SID) of IAA_lim_ with the SID of average amino acid for different foods. Results are shown in Figure 6 and indicate that although some deviations were observed, particularly in products where the IAA_lim_ was lysine, the overall trend showed a high degree of correlation (R^2^ > 0.99) with a slope very close to 1. This indicates that the use of average amino acid digestibility rather than digestibility of IAA_lim_ can lead to deviations, but is unlikely to lead to a structural over- or underestimation in dietary protein quality values. As a result, structural differences between (untruncated) PDCAAS values and DIAAS values for different protein sources appear mainly attributable to the use of faecal digestibility rather than ileal digestibility values and not from the use of total digestibility of protein rather than the digestibility of the IAA_lim_. 

In addition to ileal and faecal digestibility, as applied in DIAAS and PDCAAS methodology, respectively, there are also other methods for determining protein digestibility in vivo, which are included in Table 3. This includes several methods based on nitrogen balance, i.e., the biological value (BV) method, net protein utilization (NPU) method and the true digestibility (TD) method. In these methods based on nitrogen balances, rodents are fed with experimental diets containing the test protein as sole source of nitrogen and with a nitrogen-free diet for the control group [32]. Endogenous nitrogen losses are measured from the control group fed the nitrogen-free diet and, together with total nitrogen consumption and the nitrogen content of the excreta in the experimental group are measured. These values can further be used to calculate the protein quality [51] (Table 3). One of the fundamental assumptions of the nitrogen balance methods is that the amount of nitrogen consumed is either used or excreted without any other metabolic consequences [52]. These methods, however, overlook that there can be delay in nitrogen excretion (especially in case of a large urea pool), the metabolic contributions to the nitrogen excreted and ignore variation in digestibility of consumed protein [28,51].

### 3.3. Methods for Determining Protein Quality Based on Growth Studies 

In addition to the PDCAAS method and DIAAS method described in Section 3.2, various other methods to measure protein quality have been used and are included in Table 3 and Figure 4. These methods, however, use some notably different measurement principals, e.g., the change in body weight, net nitrogen utilization, rather than the previously described digestibility of amino acids at faecal or intestinal level [30]. Methods using change in body weight to evaluate protein quality include the protein efficiency ratio (PER) and the net protein retention/ratio (NPR) and are commonly determined compared to a reference protein, often casein [33]. The PER and NPR methods have an advantage of being relatively easy to conduct [28]. These methods involve data collection from rodent feeding trials, where growing rodents are fed either the test protein or the reference protein for a set period of time [51]. At the end of trial, the final weights of the experimental rodents from each group are determined, and weight gain is expressed relative to amount of protein consumed. The final protein quality value for the test protein is calculated relative to that of the reference protein, which is most commonly casein [60]. One drawback of these methods is that, the digestibility of sulphur-containing amino acids (SAAs) in rodents is higher than in humans, and the outcomes thus can overestimate the protein quality for humans [69]. The PER and NPR methods also overlook the role of other nutrients in the experimental diet that possibly contribute to the weight gain of the test subject [28].

### 3.4. Methods for Determining Protein Quality and Protein Digestibility In Vitro 

In addition to in vivo methods listed in Table 3, in vitro methods for determining protein digestibility and protein quality have been reported. In vitro methods simulate the digestion process in a laboratory setting to measure protein quality and these methods may resemble the digestion process in either a static or (semi-)dynamic manner. Static models do not replicate actual human digestion processes, including peristalsis and gradual introduction of different enzymes at different time, while dynamic methods can replicate these better [82]. In vitro methods, such as in vitro protein digestibility (IVPD) method, compare total protein content of the food and total digested protein, while other methods, such as in vitro protein digestibility corrected amino acid score (IVPDCAAS) method, further correct for the first limiting amino acid compared to reference protein [83]. Although in vitro analysis can be cheaper and easier methods to predict the outcome of in vivo digestibility, the complexity of in vivo digestibility has not been realized fully in an in vitro model [84]. However, development of in-vitro analysis has potential to provide insight into processing of protein during digestion. Analytical methods such as size exclusion chromatography can estimate the percentage of small peptide available for absorption. A physiologically relevant protein digestibility is estimated by combining the total dissolved protein and percentage small peptide [85]. Overall, though, data from in vivo studies remain preferred, particularly when based on ileal digestibility. Outcomes from such studies are described in Section 4. 

## 4. Protein Quality Data from DIAAS Measurements: Interpretation and Application

As outlined in Section 3, protein quality can be determined by various methods, but estimates based on ileal digestibility of individual amino acids, as done in the DIAAS method, are preferred. To illustrate the variability and the underlying reasons for this variability, we collected available DIAAS values for dietary proteins from food products or protein ingredients. The overview of available DIAAS values is shown in Table 4, which also includes the IAA_lim_ for each product and the SID of the IAA_lim_. Furthermore, the species in which SID measurements were carried out (pigs or rats) and the reference pattern against which the DIAAS values were calculated are also included in Table 4. 

Table 4 shows a wide variability in both DIAAS values (ranging from 1 to 144%) and SID of the IAA_lim_ (ranging from 13 to 101%) between the different products. The DIAAS score is essentially the product of the SID and a normalized concentration of the IAA_lim_ (expressed relative to the concentration, per g protein, of this IAA in the reference pattern), with the former, like the DIAAS score, expressed as a percentage and the latter expressed as a fraction. Hence, a normalized concentration of IAA_lim_ = 1 yields DIAAS (%) = SID (%), whereas for SID = 100%, DIAAS = normalized concentration of IAA_lim_ × 100%. Hence, a comparison of DIAAS values with SID values for IAA_lim_ provides useful insights in the relative contributions of SID and the concentration of IAA_lim_ to the DIAAS score. With only one exception (i.e., eggs) all products with a DIAAS value > 100% show a SID for the IAA_lim_ of >90%, indicating that for these products the IAA_lim_ is highly digestible and that the DIAAS value is primarily determined by the concentration of the IAA_lim_ in the protein, rather than its digestibility. For products with DIAAS scores < 100%, the comparison of SID and DIAAS values for the protein sources can also be used to identify whether SID or IAA_lim_ concentration contributed stronger to the lower DIAAS score. For almost all products with DIAAS values < 100%, it was found that SID > DIAAS (Table 4). This indicates that the impact of a low normalized concentration of IAA_lim_ contributed stronger to the deviation in DIAAS score from 100% than the SID.

When looking at the different food groups, all animal-based foods have a DIAAS value > 90%, and most even >100%, whereas of the plant-based food groups, only soy-based products and mung beans have a DIAAS value > 90% (Table 4). Hence, animal-based foods are often be considered as a source of higher quality protein than most plant-based foods [52,57]. However, next to comparing or ranking products on the basis of DIAAS values, one has to consider that food items are generally consumed in meals combining different food items, whereby the digestible amounts of individual IAAs of various food items can complement each other in terms of protein intake adequacy [89], which is discussed in Section 6.

An overview of DIAAS values and SID values based different food groups is presented in Table 5. The most commonly found IAA_lim_ in cereals and nuts is lysine, whereas valine is the most commonly-found IAA_lim_ in meat products (beef, pork), and the SAAs are the IAA_lim_ in legumes. Dairy products show either histidine (n = 4) and SAA (n = 4) as the IAA_lim_. Histidine was the IAA_lim_ for whey protein-based products (Table 4), whereas the IAA_lim_ for milk products were the SAAs, due to the dominance of caseins in these products as the main protein class. Interestingly, Table 4 and Table 5 show that not all IAAs are the IAA_lim_. The IAAs isoleucine, tryptophan and the aromatic amino acids (AAA) were not reported as the IAA_lim_ in any of the food items included in Table 4 and food groups included in Table 5. Overall, the SID of IAA_lim_ was >90% for all animal-source foods except for eggs, whereas the digestibility of IAA_lim_ of cereals varied from as low as 13% to as high as 96%. One of the reasons behind this large variation in digestibility of the IAA_lim_ for cereals comes from the inclusion of raw and processed cereals [55]; the impact of processing on digestibility is further discussed in Section 5. The digestibility of IAA_lim_ in legumes was in the range of 75–101% (Table 5). In general, it was observed that variation in DIAAS values within food groups was larger for plant-based foods than for animal-based foods.

As outlined above, DIAAS values reported are based on the IAA_lim_ [60,90]. While these metrics work well for comparing or ranking protein sources, the additional information behind the DIAAS protein quality measurement can also provide valuable information, particularly when considering complementarity of protein sources in a meal or dietary perspective. Furthermore, when an IAA is classified as the IAA_lim_ in a DIAAS value, it is important to distinguish whether this IAA is present at insufficient levels to meet requirements (i.e., DIAAS < 100%) or classified as limiting following the classification system but still meets requirements (DIAAS > 100%). For example, none of the DIAAS values that classify valine as the IAA_lim_ in pork products in Table 4 and Table 5 are limiting to the extent that they cannot meet requirements, whereas in all 23 cases where that lysine is classified as the IAA_lim_ in cereals, DIAAS values < 100% (Table 5). So, lysine as the IAA_lim_ in cereals is considered to be an absolute limiting IAA. Thus, in the context of nutritional requirements, it is important to see if the IAA_lim_ of a product is actually limiting in a nutritional context or not. 

**Figure 7:**
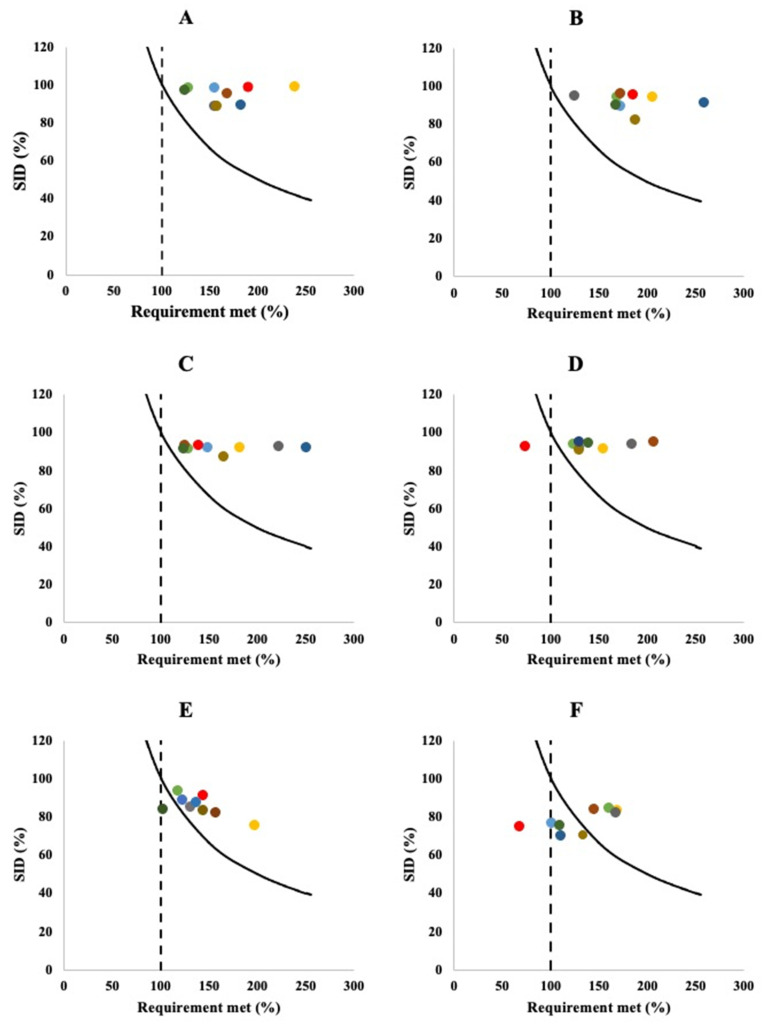
Concentration (expressed as a percentage of the recommendation for adults outlined in Table 2) and standardized ileal digestibility (SID) for histidine (●), isoleucine (●), leucine (●), lysine (●), sulphur containing amino acids (●), aromatic amino acids (●), threonine (●), tryptophan (●) and valine (●) in pork belly; **- - -**: adequacy marker for amino acid requirement; 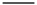: cut-off for actual requirement as a combination of amino acid content and digestibility of the respective indispensable amino acid; (**A**; data from [76]), skim milk powder (**B**; data from [57]), soy flour (**C**; data from [57]), rice (**D**; data from [80]), cooked peas (**E**; data from [87]) and maize (**F**; data from [80]).

Meeting requirements for digestible IAA levels for protein sources is a combination of concentration and digestibility; i.e., a digestibility <100% can be compensated by a higher concentration of the respective IAA present in the protein. To demonstrate this, we plotted the SID against the IAA concentrations, expressed as a percentage of the digestible IAA of the reference protein for adults (Table 2), for pork belly [79], skim milk powder [57], soy four [57], rice [80], cooked peas [87] and maize [80] in Figure 7. This figure includes a line of sufficiency, indicating that combinations of IAA concentration and SID of the IAA meet the minimal requirement for the IAA. Scores of an IAA above this line thus meet requirements, whereas IAAs falling below the line do not meet requirements.

When considering the products shown in Figure 7, different product clusters can be identified based on the adequacy of number of IAAs. For pork belly, skim milk powder, and soy flour, digestible levels of all IAAs were above the requirements, which translates into DIAAS values > 100%. However, DIAAS values are calculated based on the IAA_lim_ and fails to highlight that other IAAs are present in further excess, and could thus act as complimentary supply for other protein sources lacking in specific amino acids. For instance, the large excess of digestible lysine in skim milk powder (Figure 7) can be important for compensation for deficiencies in many other proteins lacking digestible lysine, e.g., cereals [77].

The other three products in Figure 7, rice, cooked peas and maize, show a different pattern. Rice is a clear example of a protein source where one specific IAA, in this case lysine, is strongly lacking, whereas the other IAAs are present at (near-)sufficient amounts in digestible form (Figure 7). Improving digestibility of protein in rice could improve the score for lysine, but would still not reach 100% of the requirement, because the concentration of lysine present in rice protein is simply insufficient to meet requirements even at 100% digestibility. Protein sources with excess digestible lysine, however, could thus complement rice protein, thereby providing a complete set of IAAs at adequate levels in a mixture. Cooked peas are another example of a food with single amino acid in inadequate levels in digestible form; however, in this case, improving digestibility could actually lead to meeting requirements (Figure 7). While only one IAA is clearly lacking in cooked peas, five other IAAs (leucine, isoleucine, tryptophan, threonine and AAA) are just adequate to meet human requirement, thus not capable to contribute to complement other food items lacking these amino acids. For maize, lysine is the first limiting amino acid, with both concentration and digestibility low; however, threonine, tryptophan, isoleucine, and valine are also well below requirements due to low digestibility (Figure 7).

Figure 7 thus shows the importance of considering all IAAs and their digestibility when considering protein quality, particularly when using protein quality metrics to look beyond individual protein sources, and consider them in the context of human nutrition, where people consume meals and diets consisting of multiple protein sources. While some IAAs (AAA, isoleucine, tryptophan) were not observed to be first limiting amino acid, these IAAs can be a second limiting amino acid, which can also be below the level of requirement. For example, tryptophan is the second limiting IAA, after lysine, with DIAAS value for this IAA of <71% in maize and maize [77,80]. Therefore, looking only at the IAA_lim_ does not always provide information on the available amino acid to compensate for limiting IAAs when combined in a meal with other food/protein sources.

## 5. Influence of Food Processing and Preparation on Protein Quality

When considering protein quality in the perspective of the human diet, it is important to keep in mind that all food items consumed will have undergone some sort of processing. This may be very mild, e.g., washing or removal of inedible materials [91], but also much more extensive, e.g., the isolation of a protein source from a raw material followed by incorporation it as an ingredient in another food product which can receive multiple processing steps [92]. Furthermore, before consumption a food product may also be subjected to additional processing in the form of boiling, baking, frying, grilling, or steaming [93]. These processing steps can notably effect on protein quality. Table 4 already showed that different beef and pork preparation methods affect DIAAS values. Furthermore, baking reduced the DIAAS value of buckwheat, whereas extrusion increased it [83], cooked pinto beans had a higher DIAAS value than baked pinto beans [94] and microwaved cowpeas had a higher DIAAS value after autoclaving then after roasting [95].

Processing can affect DIAAS values through effects on the digestibility of the protein, as well as through changes in the concentrations of available IAAs. In this section, we will discuss the influence of processing on protein quality via effects on IAAs and protein digestibility. For this, we will focus on processing techniques that can be applied which will not change protein composition of a product; e.g., the fractionation of proteins from a single source, which would be considered processing as well, is outside the scope of this paper.

### 5.1. Influence of Processing-Induced Modifications in Amino Acids on Protein Quality

During processing, particularly at high temperature, amino acids can undergo various chemical reactions, as a result of which they may become nutritionally unavailable [96]. The most commonly observed example of this is the Maillard reaction, which involves the conjugation of lysine with reducing sugar, leads to formation of blocked (or glycated) lysine, whose metabolic availability is reduced [97,98,99]. Reduced lysine absorption was shown for milk protein powder in which 20 or 50% of lysine was glycated, compared with the same powder in which only 3% of lysine was glycated [99]. Reduced absorption of lysine was also shown from skim milk that had 50% blocked lysine compared to skim milk without blocked lysine [97]. This latter study also showed that the net total amino acid absorption over 12 h postprandial was 15% lower from the milk with 50% blocked lysine [97]. This effect on total amino acid absorption is larger than what would be expected solely from lysine and thus reflects reduced absorption of other amino acids, probably due to the fact that glycation of lysine can also reduce the cleavage of peptide bonds in polypeptides and proteins by digestive enzymes in the vicinity of the blocked lysine residue [100]. 

Thermal treatment has been associated with formation of blocked lysine in many products, e.g., rapeseed meal [98], dairy products [101] and cereal-based products [102]. The amount of blocked lysine varies based on the processing method, duration and storage conditions. Blocked lysine levels up to 20% of total lysine have been reported in cereal products [103] due to processing, while up to 14% lysine was blocked for some dairy products [103]. While lysine is not a limiting amino acid in dairy products [57,72,77], it is the first limiting amino acid in wheat products [72,80,81], thus strongly impacting protein quality. In addition to lysine, the overall amino acid bioavailability can also be impacted by processing-induced modification of other amino acids. Processing steps involving heat and alkaline treatment can result in losses or reduced digestibility of not only lysine, but also cysteine, threonine and/or total protein [104]. These processing techniques can induce racemization of L-amino acids and the formation of crosslinked peptides. These cross-links not only reduce amino acid availability, but also hinder the enzyme activity and in turn reduce the digestibility [105]. For example, alkali-treated soy protein had lower digestibility (83%) compared to soy protein that was heat treated (97%) [106]. Effects of processing on protein digestibility are further discussed in Section 5.2. 

### 5.2. Influence of Processing-Induced Changes in Protein Digestibility on Protein Quality

In addition to through processing-induced amino acid modifications, processing can also affect protein quality values for dietary proteins through changes in protein digestibility. An extensive overview of the effect of processing on protein digestibility is shown in Table 6. From this table, it is clear that processing can result in both large increases and decreases in protein digestibility. Microwaving eggs increased protein digestibility by 40% [107], and autoclaving (110 °C for 45 min) soy beans improved protein digestibility by 38% [108], whereas roasting (230 °C) Bambara groundnuts reduced protein digestibility by 37% [109]. Dry roasting tended to reduce protein digestibility in many products (Table 6), whereas all foods, except sorghum flour [110] and Bambara ground nuts [109], showed increased protein digestibility after wet thermal treatments, such as boiling or autoclaving. For almost all animal-source foods, processing increases the overall protein digestibility (Table 6). However, for cooking of ground beef [76] and roasting of topside steak [78], these increases in overall protein digestibility, resulted in a reduction, rather than an increase, in DIAAS because of a reduction in the digestibility of the IAA_lim_ for these products, leucine and valine respectively. This highlights the importance to look at the individual amino acid digestibility along with overall protein digestibility to understand the role of processing in influencing protein quality of a food product.

Processing-induced changes in protein digestibility can arise from effects on protein structure, anti-nutritional factors, accessibility for the digestive enzymes, but also through effects on the non-protein constituents [55]. For example, processing can affect protein structure and therewith, digestibility [117]. Furthermore, naturally occurring anti-nutritional factors, such as protease inhibitors (e.g., trypsin-chymotrypsin inhibitor), polyphenols (e.g., tannins), phytate and non-starch polysaccharides present in various plant-based dietary protein sources impact protein digestibility [55] and have been demonstrated to be reduced by processing, leading to improved protein digestibility [118]. e.g., the boiling of peas reduces the protease inhibitor activity [119], whereas extrusion of fava and kidney bean reduces the phytic acid and tannin content [120]. Protein hydrolysis is also widely used in food industry and can improve their digestibility, e.g., for milk protein [121] and plant protein [122]. Processing can also improve protein digestibility through changes in non-protein components in the food. For instance, cell wall degradation can increase accessibility to the protein for digestive enzymes and increase protein digestibility [123]. Furthermore, particle size reduction via milling, grinding or mincing increases the surface area of the food and hence can increase exposure to digestive enzymes. Studies have shown that milling increased protein digestibility in soybean meal [124] and lupin [125] and that amino acids from minced meat are more rapidly absorbed compared to those from the original steak that the minced meat was prepared from [126].

Overall, it is clear that processing can have a large effect on protein quality via both effects on amino acids and effects on protein digestibility. As a results, protein quality measures on raw materials and/or ingredients cannot necessarily be taken as representative for food products that are actually consumed.

## 6. Complementarity of Different Protein Sources 

Previous sections in this review have described the concept of protein quality and its importance in ensuring the dietary intake of proteins meets bodily requirements for both IAAs and nitrogen. Typically, human requirements for protein intake are defined as an RDA, in grams of protein per person per kg of bodyweight per day [27,30]. This recommendation, however, refers to a protein source which contains a complete profile of IAAs and is fully digestible (i.e., DIAAS value ≥ 100) [27]). As discussed in Section 4 and shown in Table 4, many proteins do not meet these criteria, due to the lack of one or more IAAs in adequate quantities or due to low digestibility. However, it is also clear from Section 4 and Figure 7 that many proteins, even those with DIAAS values < 100, contain one or more IAAs in digestible form at concentrations above the requirements. While this latter parameter is not typically included in protein quality metrics, it is key to consider in a dietary perspective because human protein consumption is mostly based on a mixture of different foods in meals throughout the day, and thus often a mixture of different proteins within a meal [127]. 

The digestible IAA contents of dietary proteins in a meal are considered to be additive in nature and can be used to calculate the final protein quality of a meal [77]. As a result, the content of digestible IAA in the different foods in a meal may complement each other to improve the overall IAA content of the meal [30]. Lysine is recognized as the IAA_lim_ in most cereals (Table 5; [123]) and also in the diet of the general population in India, Sub-Saharan African and China [21]. However, interpreting protein quality on a dietary level is a topic that warrants care and consideration. Whereas for other nutrients, e.g., vitamin A, intake on a daily or weekly basis can be summed [128], the time scales for protein utilization are much shorter. This due to the fact that the human body does not keep storage of protein or IAAs, like it does for many micronutrients and for energy, e.g., in the liver, in tissue or in the skeleton. Studies on postprandial aminoacidemia show a rise in blood amino acids after food consumption, and this rise subsiding again back to basal levels within a few hours after consumption [129,130,131]. Within this time frame, the amino acids taken up are metabolically utilized in the synthesis of proteins and materials by the body [132]. Amino acids that are not metabolized are oxidized [133,134]. The extent of oxidation of absorbed amino acids is primarily driven by the amount of amino acids absorbed, because excess absorption beyond metabolization capacity will lead to oxidation of non-metabolized amino acids [133]. Furthermore, oxidation of amino acids is also determined by the relative ratio compared to the required amino acid profile. Oxidation of the out of balance amino acids absorbed will occur because they cannot be utilized [135].

Studies have also shown that overconsumption of protein results in inefficient use of protein, because of oxidation of excess amino acids [136]. In this respect, it is important to realize that protein consumption is often skewed to a specific time of the day [137,138,139]. Studies in Japan and the USA have shown that the distribution of protein consumption is uneven throughout the day: >40% (~32 g) of the protein is consumed at dinner, while protein consumption at breakfast is <20% of daily protein intake [137,138]. This asymmetric distribution of protein intake per meal might result in lower efficiency of muscle protein synthesis and higher amino acids oxidation after the meal with excess protein consumption [140]. Hence, skewed distribution of protein intake throughout the day entails the risk of suboptimal protein utilization due to overconsumption at certain moment.

Suboptimal protein utilization can also occur due to an unfavourable composition of the absorbed amino acids at a given time point, i.e., when the IAA ratios differ from those shown in Table 2. If one or more of the IAAs are lacking, the other IAAs cannot be utilized either and will eventually be oxidized [141]. Hence, supply of IAA needs to be synchronous for optimal utilization. This was exemplified clearly in a study in growing calves, where the synchronous and asynchronous supply the IAAs lysine and threonine throughout the day was studied [142]. Utilization of protein was found to be significantly higher in calves which were fed all IAAs at the same time, compared to calves which were fed the IAAs in an asynchronous manner [142]. This also applies to humans, i.e., maximal utilization requires the IAAs to be present at the right ratio in the blood at same time, combined with sufficient DAAs [143]. Considering the kinetics of postprandial aminoacidemia, where blood amino acid levels return to basal levels after several hours [129], this essentially requires balanced intake of digestible IAA over the course of a meal, because timing between meals is too long and any residual IAA not utilized would have become oxidized. 

A balanced IAA composition can be derived from a single product (i.e., from products with DIAAS > 100), but also from a mixture of products, for which digestible IAA composition from the different products can complement each other. For this, however, it is important that complementarity is considered at a meal basis, and not at the basis of daily, weekly or monthly diet. In other words, lysine deficiency in cereal-based breakfast products can be compensated by consuming the products together with a protein source with a surplus of digestible lysine, e.g., milk [77], but only when consumed together in a meal. As shown in Table 4 and Table 5, most cereals are limiting in lysine, whereas legumes mainly have SAA as the IAA_lim_. If legumes are combined with cereals, it can potentially improve the overall quality of protein intake per combination to some extent [144,145] However, it was seen that major contributor of global protein supply for the year 2018 were cereals (~22g protein/capita/day), whereas legumes contributed less (~2 g protein/capita/day) in global protein supply [146].

**Figure 8:**
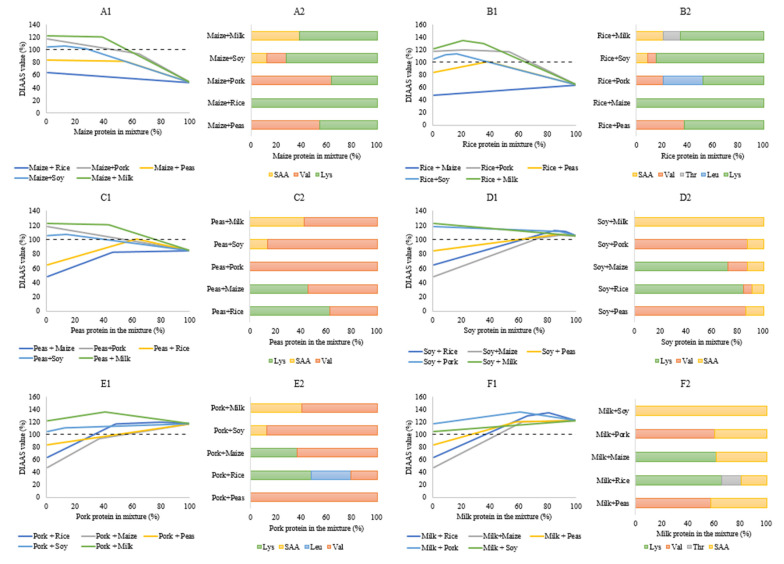
DIAAS value (**A1**,**B1**,**C1**,**D1**,**E1**,**F1**) and first limiting indispensable amino acid (**A2**,**B2**,**C2**, **D2**,**E2**,**F2**) for mixtures of protein from maize (**A1**,**A2**; data from [80]), rice (**B1**,**B2**; data from [80]), cooked peas (**C1**,**C2**; data from [87]), soy flour (**D1**,**D2**; data from [57]), pork belly (**E1**,**E2**; data from [76]) and skim milk powder (**F1**,**F2**; data from [57]) with any of the other 5 foods; Dashed line in (**A1**, **B1**, **C1**, **D1**, **E1**, **F1**) indicates a DIAAS value of 100%.

The DIAAS values of different food combinations can be predicted using the values from the respective foods in combination [77]. To illustrate complementarity of binary mixtures of proteins, we calculated complementarity in terms of protein quality for combinations of dietary protein from maize [80], rice [80], cooked peas [87], soy flour [57], pork belly [79] and skim milk powder [57], based on digestible IAA levels present in the protein in these products. For each combination of two foods, mixtures from 0 to 100% protein from each source at 1% intervals were considered, whereby digestible IAA levels for the mixtures were calculated and compared to the reference IAA composition for adults (Table 2). Based hereon, the DIAAS value of mixture was calculated and the IAA_lim_ for each mixture at each ratio was identified. Results from these calculations (Figure 8), clearly show that for many combinations of two different foods, compensation for lacking IAAs in one food by those present in another food is possible. For example: rice, maize and cooked peas each have a DIAAS value < 100% individually, but when they are mixed with milk, soy and pork, mixtures with DIAAS values > 100% can be achieved (Figure 8). However, there are also cases where combinations of foods do not compliment fully: the combinations of rice + maize (Figure 8A1,B1) and peas + maize (Figure 8A1,C1), DIAAS values for the mixtures were higher than for the individual protein sources but remained <100% at all ratios.

In addition to the ability of mixtures of two foods to yield a DIAAS value > 100%, the ranges of the ratios within which this can be achieved are also important to consider, because in some cases, these margins are very narrow. For example, mixing peas and rice can yield a DIAAS value > 100%, but only at a ratio of 60–65% of protein from peas and 35–40% of protein from rice (Figure 8B1,C1). When looking at the capacity of compensation for individual foods, the highest ratio required to yield DIAAS values > 100% was with any other product was 45% for protein from milk, whereas for protein from pork and soy it was 51% and 70%, respectively (Figure 8). Some combinations show a linear change in DIAAS value with the increase or decrease of food items such as the combinations maize + rice, pork + peas, and milk + soy (Figure 8). This can be explained by the fact that both of these foods in the combination have same first limiting amino acid and therefore the degree of compensation is in line with the capacity of the food to meet human requirement and its concentration in the combination.

In addition to considering overall score of the protein mixtures, the amino acid that was limiting at each ratio in the mixture is also shown in horizontal bar charts in Figure 8. In these plots, scenarios can be distinguished wherein one, two or three different IAAs are indicated as IAA_lim_ over the range of ratios tested. For mixtures of pork and peas, valine was the limiting amino acid at all ratios, whereas for milk and soy, the SAA were always limiting. For many other combinations, two IAAs were found to be limiting over the range, i.e., the IAA_lim_ for each of the two protein sources in the mixture. Interestingly, however, there were also a few instances where not only the first limiting amino acids of the two foods in the combination, but also a third limiting IAA was detected to be most limiting in the combination at specific concentrations of individual foods (Figure 8). For instance, at certain ratios, threonine was the most limiting IAA for the combination of milk and rice, whereas the SAAs are first limiting in milk protein and lysine is first limiting in rice protein. Likewise, for combinations of pork + rice (leucine), soy + rice (valine), and soy + maize (valine) an IAA was limiting at certain ratio which was not the IAA_lim_ in one of the products. This ‘new’ limiting IAA was the second limiting amino acid for one of the foods in the combination. This true complementarity of proteins from different foods would not be seen if only the most limiting amino acid was studied and highlights the importance of looking at individual amino acids rather than focusing only on the most limiting amino acid. 

Combining foods with different limiting amino acid can complement the lacking amino acid to improve the overall protein quality of the combination. However, as seen in the examples not all combinations can improve the overall quality fully. Therefore, the capacity of foods included in a combination to complement for the quality of the combination should be taken into consideration. While we considered binary mixtures for this review, human diet can assuredly comprise of multiple foods in combination at meals. The compensatory behaviour of these foods and protein content can express complex behaviour to compensate for the lacking amino acid in the mixture. Thus, careful combination of foods to complement lacking amino acids while considering the time of consumption has the potential to assure adequate intake of protein that efficiently meets the human requirement of protein as well as IAAs.

## 7. Conclusions and Future Perspectives

The importance of healthy and sustainable diets that provide all nutrients without imposing negative effects of human health or planetary health is clear. Routes to achieve such healthy and sustainable diets remain under debate. One of the nutrients most discussed in this perspective is protein, because the ability to contribute to human requirements differs widely between dietary proteins, due to differences in amino acid composition as well as due to differences in digestibility. Both these factors differ between protein sources, but are also affected by processing. Methods to determine protein quality, based on digestibility an amino acid composition of dietary proteins have been designed, the preferred one of which is the DIAAS method, which is based on ileal digestibility of protein. In the context of health and sustainable diets, however, it is crucial to not only consider DIAAS values for proteins, which are based on the IAA_lim_, but look beyond this and focus on all IAAs in the protein. Otherwise, a key factor of complementarity of dietary protein sources is overlooked and protein needs are likely to be overestimated. The inclusion of protein quality in the design of healthy and sustainable diets thus requires consideration of all IAAs. Furthermore, while complementarity of protein sources is key, it should be considered at the right time scale, i.e., at the meal level and not the dietary level. The human protein metabolism does not complementarity at longer time scales, due to oxidation of excess amino acids. Overall, it is clear that the consideration of protein quality in healthy and sustainable diets is of high importance, but that such considerations should be based not on a product level, but on a meal level, requiring data integration. 

## Figures and Tables

**Table 1:** Recommended intake of indispensable amino acids (in mg/kg body weight/day) for humans in different age groups (data from [27,30,32]).

Age (Years)	His	Ile	Leu	Lys	SAA *	AAA **	Thr	Trp	Val
0.5–1	22	36	73	64	31	59	34	9.5	49
1–2	15	27	54	45	22	40	23	6.4	36
3–10	12	23	44	35	18	30	18	4.8	29
11–14	12	22	44	35	17	30	18	4.8	29
15–18	11	21	42	33	16	28	17	4.5	28
>18	10	20	39	30	15	25	15	4.0	26

* SAA = Sulphur-containing amino acids (Cys + Met); ** AAA = Aromatic amino acids (Phe + Tyr).

**Table 2:** Recommended reference pattern for indispensable amino acids (in mg/g protein) for humans in different age groups (data from [30,32]).

Age (Years)	His	Ile	Leu	Lys	SAA *	AAA **	Thr	Trp	Val
0–0.5	21	55	96	69	33	94	44	17	55
0.5–3	20	32	66	57	27	52	31	8.5	43
>3	16	30	61	48	23	41	25	6.6	40

* SAA = Sulphur-containing amino acids (Cys + Met); ** AAA = Aromatic amino acids (Phe + Tyr).

**Table 3:** Overview of in vivo methods used for determining protein quality and protein digestibility.

Method	Measurement Principle	Calculations	Refs.
Protein quality methods			
Protein efficiency ratio (PER)	Ratio of weight gain and protein consumed by test group over control (preferred reference protein: casein)	$\frac{\left(Weight\;gain\;\left(g\right)\;TP\;/Amount\;of\;protein\;consumed\;\left(g\right)\;TP\right)}{\left(Weight\;gain\;\left(g\right)\;RP\;/Amount\;of\;protein\;consumed\;\left(g\right)\;RP\right)}$	[51]
Net protein ratio (or net protein retention) (NPR)	Difference in weight gain between a test protein group and protein-free diet group per gram of protein consumed by the test protein group.	$\frac{Weight\;change\;test\;group\;\left(g\right)\;-\;Weight\;change\;of\;protein\;free\;diet\;group\;\left(g\right)}{Protein\;consumed\;\left(g\right)}$	[58]
Protein digestibility corrected amino acid score (PDCAAS)	Ratio of IAA_lim_ in test protein compared to reference protein corrected for faecal protein digestibility	$\left[\frac{IAA_{lim}\;in\;TP\;\left(mg/g\;TP\right)}{IAA_{lim}in\;RP\;\left(mg/g\;RP\right)}\right]\times Faecal\;digestibility\;TP\%$	[32,51,59]
Digestible indispensable amino acid score (DIAAS)	Ratio of IAA_lim_ in test protein compared to reference protein corrected for ileal digestibility of IAA_lim_	$\left[\frac{IAA_{lim}\;in\;TP\;\left(mg/g\;TP\right)}{IAA_{lim}in\;RP\;\left(mg/g\;RP\right)}\right]\times Ileal\;digestibility\;IAA_{lim}\%$	[30,60]
Protein digestibility methods			
True Digestibility (TD)	Percentage of nitrogen observed from protein (food) consumed in the GI tract	$\frac{N\;intake\;test\;group\;\left(g\right)\;-\;\left(Faecal\;N\;test\;group\;-\;Endeginous\;faecal\;N\right)\;\left(g\right)}{N\;intake\;test\;group\;\left(g\right)}\times 100$	[51]
Biological value (BV)	Retained nitrogen over total nitrogen intake, with corrections for faecal and urinary losses.	$\frac{N\;intake\;TP\;-\;\left(Faecal\;N\;-\;Fecal\;N\;on\;N\;free\;diet\right)\;-\;\left(Urinary\;N\;-\;Urinary\;N\;on\;N\;free\;diet\right)}{N\;intake\;of\;TP\;-\;\left(Fecal\;N\;-\;Fecal\;N\;on\;N\;free\;diet\right)}\times 100$	[58]
Net protein utilization (NPU)	Retained nitrogen over total nitrogen intake, with corrections for faecal and urinary losses.	$\frac{N\;intake\;TP\;-\;\left(Faecal\;N\;-\;Faecal\;N\;of\;N\;-\;free\;diet\right)\;-\;\left(Urinary\;N\;-\;Urinary\;N\;on\;N\;-\;free\;diet\right)}{N\;intake\;of\;TP}\times 100$	[32]
Dual isotope tracer method	Compares AA in circular system from intrinsically labelled test protein consumed together with a reference protein with known digestibility labelled differently	$\frac{plasma\;AA\;\left(H\;labelled\;TP\right)\;÷\;meal\;AA\;\left(H\;labelled\;TP\right)}{plasma\;AA\;\left(C\;labelled\;RP\right)\;÷\;meal\;AA\;\left(C\;labelled\;RP\right)}\times 100\times Digestibility\;of\;RP\times Transamination\;correction\;factor$	[61,62]

Abbreviations: TP = Test Protein; RP = Reference Protein; AA = amino acids; N = Nitrogen, IAA = indispensable amino acid IAA_lim_ = first limiting indispensable amino acid.

**Table 4:** Overview of digestible indispensable amino acid score (DIAAS) values, including the first limiting indispensable amino acid (IAA_lim_) and its standardized ileal digestibility (SID) as well as the species in which testing was performed and the protein reference pattern against which DIAAS was calculated for different food items. Items are ranked from highest to lowest DIAAS value ^1^.

Food Item	Food Group	DIAAS Value (%)	IAA_lim_	SID of IAA_lim_ (%)	Test Species	Protein Reference Pattern	References
Dry milk	Dairy	144	SAA	94	Pig	>3-year-old	[77]
Bacon (smoked-cooked)	Pork	142	Valine	95	Pig	>3-year-old	[79]
Milk protein concentrate	Dairy	141	SAA	101	Pig	>3-year-old	[57]
Pork loin (medium)	Pork	139	Valine	95	Pig	>3-year-old	[79]
Whey protein concentrate	Dairy	133	Histidine	97	Pig	>3-year-old	[57]
Ham (alternatively-cured)	Pork	133	Valine	95	Pig	>3-year-old	[79]
Ribeye (roast, medium)	Beef	130	Valine	95	Pig	>3-year-old	[76]
Bologna	Pork	128	Leucine	97	Pig	>3-year-old	[76]
Ham (conventionally-cured)	Pork	126	Valine	96	Pig	>3-year-old	[79]
Whey protein isolate	Dairy	125	Histidine	100	Pig	>3-year-old	[57]
Ham (non-cured)	Pork	124	Valine	93	Pig	>3-year-old	[79]
Skimmed milk powder	Dairy	123	SAA	99	Pig	>3-year-old	[57]
Egg	Egg	122	SAA	75	Pig	>3-year-old	[86]
Ground beef (raw)	Beef	121	Leucine	99	Pig	>3-year-old	[76]
Beef jerky	Beef	120	SAA	98	Pig	>3-year-old	[76]
Salami	Pork	120	Valine	96	Pig	>3-year-old	[76]
Pork belly (raw)	Pork	119	Valine	97	Pig	>3-year-old	[79]
Milk protein concentrate	Dairy	118	SAA	94	Rat	0.5–3-year-old	[72]
Pork loin (medium-well done)	Pork	118	Valine	95	Pig	>3-year-old	[79]
Bacon (smoked)	Pork	117	Valine	95	Pig	>3-year-old	[79]
Pork loin (well-done)	Pork	117	Valine	95	Pig	>3-year-old	[79]
Ribeye (roast, medium-rare)	Beef	111	Valine	97	Pig	>3-year-old	[76]
Whey protein isolate	Dairy	109	Histidine	99	Rat	0.5–3-year-old	[72]
Ribeye (well-done)	Beef	107	Valine	97	Pig	>3-year-old	[76]
Soy flour	Legumes	105	SAA	101	Pig	>3-year-old	[57]
Ground beef (cooked)	Beef	99	Leucine	97	Pig	>3-year-old	[76]
Topside steak (boiled)	Beef	99	Valine	99	Pig	>3-year-old	[78]
Topside steak (pan fried)	Beef	98	Valine	98	Pig	>3-year-old	[78]
Soy protein isolate	Legumes	98	SAA	98	Pig	>3-year-old	[57]
Whey protein concentrate	Dairy	97	Histidine	98	Rat	0.5–3-year-old	[72]
Topside steak (raw)	Beef	97	Valine	98	Pig	>3-year-old	[78]
Mung beans (cooked)	Legumes	94 ^2^	Threonine	77	Pig	>3-year-old	[87]
Topside steak (roasted)	Beef	91	Valine	98	Pig	>3-year-old	[78]
Soy protein isolate	Legumes	91	SAA	94	Rat	0.5–3-year-old	[72]
Soy protein isolate	Legumes	90	SAA	92	Rat	0.5–3-year-old	[72]
Peas (cooked)	Legumes	88 ^2^	Valine	87	Pig	>3-year-old	[87]
Broad beans (cooked)	Legumes	87 ^2^	Valine	91	Pig	>3-year-old	[87]
Pistachio (raw)	Nuts	86	Lysine	87	Pig	>3-year-old	[74]
Pistachio (roasted)	Nuts	83	Lysine	77	Pig	>3-year-old	[74]
Pea protein concentrate	Legumes	82	SAA	95	Rat	0.5–3-year-old	[72]
Topside steak (grilled)	Beef	80	Valine	97	Pig	>3-year-old	[78]
Adzuki beans (cooked)	Legumes	78 ^2^	SAA	87	Pig	>3-year-old	[87]
Dehulled oats	Cereals	77	Lysine	85	Pig	>3-year-old	[80]
Kidney beans (cooked)	Legumes	74 ^2^	SAA	68	Pig	>3-year-old	[87]
Pea protein concentrate	Legumes	73	SAA	78	Pig	>3-year-old	[57]
Chickpeas (cooked)	Legumes	71 ^2^	Valine	83	Pig	>3-year-old	[87]
Buckwheat (cooked)	Cereals	68	SAA	86	Rat	0.5–3-year-old	[81]
Quick oats	Cereals	67	Lysine	83	Pig	>3-year-old	[77]
Oat protein concentrate	Cereals	67	Lysine	86	Pig	>3-year-old	[88]
Polished white rice	Cereals	64	Lysine	92	Pig	>3-year-old	[80]
Rice (cooked)	Cereals	60	Lysine	92	Rat	0.5–3-year-old	[72]
Kidney beans (cooked)	Legumes	59	SAA	75	Rat	0.5–3-year-old	[72]
Peas (cooked)	Legumes	58	SAA	89	Rat	0.5–3-year-old	[72]
Rolled oats (cooked)	Cereals	54	Lysine	84	Rat	0.5–3-year-old	[72]
Nutridense maize	Cereals	54	Lysine	79	Pig	>3-year-old	[80]
Dehulled barley	Cereals	51	Lysine	74	Pig	>3-year-old	[80]
Yellow dent maize	Cereals	48	Lysine	75	Pig	>3-year-old	[80]
Rey	Cereals	47	Lysine	67	Pig	>3-year-old	[80]
Tartary buckwheat (cooked)	Cereals	47	SAA	72	Rat	0.5–3-year-old	[81]
Peanuts (roasted)	Legumes	43	Lysine	92	Rat	0.5–3-year-old	[72]
Wheat	Cereals	43	Lysine	73	Pig	>3-year-old	[80]
Oats (cooked)	Cereals	43	Lysine	83	Rat	0.5–3-year-old	[81]
Brown rice (cooked)	Cereals	42	Lysine	93	Rat	0.5–3-year-old	[81]
Wheat bran	Cereals	41	Lysine	73	Rat	0.5–3-year-old	[72]
Rice protein concentrate	Cereals	37	Lysine	86	Rat	0.5–3-year-old	[72]
Polished rice cooked	Cereals	37	Lysine	92	Rat	0.5–3-year-old	[81]
Sorghum	Cereals	29	Lysine	69	Pig	>3-year-old	[80]
Whole wheat (cooked)	Cereals	20	Lysine	96	Rat	0.5–3-year-old	[81]
Cornflakes	Cereals	19	Lysine	78	Pig	>3-year-old	[77]
Adlay (cooked)	Cereals	13	Lysine	90	Rat	0.5–3-year-old	[81]
Foxtail millet (cooked)	Cereals	10	Lysine	88	Rat	0.5–3-year-old	[81]
Proso millet (cooked)	Cereals	7	Lysine	96	Rat	0.5–3-year-old	[81]
Corn-based breakfast cereal	Cereals	1	Lysine	13	Rat	0.5–3-year-old	[72]

SAA = Sulphur-containing amino acids (cysteine + methionine); ^1^ Only studies are considered where reported DIAAS values were calculated based on determination of standardized ileal digestibility and amino acid composition on the same material. Calculated DIAAS values based on calculations with data from different studies was not considered. ^2^ DIAAS values are calculated from data provided and not the reported DIAAS values in the publication based on discrepancies between published data and reported DIAAS values and communications with the authors.

**Table 5:** Overviewed of the range for digestible indispensable amino acid score (DIAAS) values, the first limiting indispensable amino acid (IAA_lim_) and its standardized ileal digestibility (SID) for different food groups. For IAA_lim_, data in brackets indicate total amount of times the IAA was IAA_lim_ in the food group, followed by the number of occurrences for which this was for a product with DIAAS < 100 and the number of occurrences for which this was for a product with DIAAS > 100. Data from Table 4.

Food Group	Number of Food items	DIAAS Value (Range)	SID IAA_lim_ (Range)	IAA_lim_
Beef	11	80–130	95–99	Valine (n = 8/5/3), Leucine (n = 2/1/1), SAA * (n = 1/0/1)
Cereals	25	1–77	13–96	Lysine (n = 23/23/0), SAA * (n = 2/2/0)
Dairy	8	97–144	94–101	SAA (n = 4/0/4), Histidine (n = 4/1/4)
Legumes	15	43–105	75–101	SAA * (n = 10/9/1) Valine (n = 3/3/0) Lysine (n = 1/1/0) Threonine (n = 1/1/0)
Pork	11	117–142	93–97	Valine (n = 10/0/10) Leucine (n = 1/0/1)
Egg	1	122	75	SAA * (n = 1/0/1)
Nuts	2	83–86	77–87	Lysine (n = 2/2/0)
All	73	1–144	13–101	Lysine (n = 26/26) Valine (n = 8/21) SAA * (n = 11/18) Histidine (n = 1/4) Leucine (n = 1/3) Threonine (n = 1/1)

* SAA: Sulphur-containing amino acids (cysteine + methionine).

**Table 6:** Overview of the effects of food processing on protein digestibility.

Food	Processing	Processing Conditions	Protein Digestibility (%)		Digestibility Method	References
			Raw	Processed		
Eggs	Microwave	-	51	91	TD	[107]
Ground beef	Cooking	Fully cooked, 72 °C	104	105	SID (pig)	[76]
Topside steak	Boiling	boiled at 80 °C completely submersed in water	97	98	SID (pig)	[78]
	Grilling	225 °C, internal temperature 35.5 °C	97	96	SID (pig)	
	Frying	186 °C, internal temperature 35.5 °C	97	98	SID (pig)	
	Roasting	oven roasting at 160 °C	97	98	SID (pig)	
Canadian cowpea	Soaking	room temp 1:5 (*w*/*v*) seed to water, 18 h	83	87	IVPD	[111]
	Boiling	35 min	83	98	IVPD	
	Roasting	180 °C for 15 min	83	78	IVPD	
	Autoclaving	15 lb pressure and 121 °C for 20 min	83	90	IVPD	
	Microwave	1200 Watt for 15 min	83	93	IVPD	
	Micronization	90 °C with 115V infrared for 2.5 min	83	80	IVPD	
Egyptian cowpea	Soaking	room temp 1:5 (*w*/*v*) seed to water, 22 h	82	87	IVPD	[111]
	Boiling	35 min	82	97	IVPD	
	Roasting	180 °C for 15 min	82	77	IVPD	
	Autoclaving	15lb pressure and 121 °C for 20 min	82	90	IVPD	
	Microwave	1200 Watt for 15 min	82	92	IVPD	
	Micronization	90 °C with 115V infrared for 2.5 min	82	79	IVPD	
Canadian kidney bean	Soaking	room temp 1:5 (*w*/*v*) seed to water, 18 h	71	76	IVPD	[111]
	Boiling	45 min	71	87	IVPD	
	Roasting	180 °C for 20 min	71	65	IVPD	
	Autoclaving	15 lb pressure and 121 °C for 20 min	71	79	IVPD	
	Microwave	1200 Watt for 20 min	71	82	IVPD	
	Micronization	90 °C with 115 V infrared for 3 min	71	68	IVPD	
Egyptian kidney bean	Soaking	room temp 1:5 (*w*/*v*) seed to water, 20 h	78	83	IVPD	[111]
	Boiling	boiled for 45 min	78	94	IVPD	
	Roasting	180 °C for 20 min	78	73	IVPD	
	Autoclaving	15 lb pressure and 121 °C for 20 min	78	86	IVPD	
	Microwave	1200 Watt for 20 min	78	89	IVPD	
	Micronization	90 °C with 115 V infrared for 3 min	78	75	IVPD	
Canadian pea	Soaking	room temp 1:5 (*w*/*v*) seed to water, 18 h	78	84	IVPD	[111]
	Boiling	boiled for 35min, 1:5 (*w*/*v*)	78	94	IVPD	
	Roasting	180 °C for 15 min	78	73	IVPD	
	Autoclaving	15 lb pressure and 121 °C for 20 min	78	87	IVPD	
	Microwave	1200 Watt for 15 min	78	89	IVPD	
	Micronization	90 °C with 115V infrared for 2.5 min	78	76	IVPD	
Egyptian pea	Soaking	room temp 1:5 (*w*/*v*) seed to water, 20 h	80	85	IVPD	[111]
	Boiling	pre-soaked (4 h) seeds boiled for 35min, 1:5 (*w*/*v*)	80	96	IVPD	
	Roasting	sandbathe at 180 °C for 15 min	80	75	IVPD	
	Autoclaving	15 lb pressure and 121 °C for 20 min	80	88	IVPD	
	Microwave	with 1:5 (*w*/*v*) water at 1200 Watt for 15 min	80	91	IVPD	
	Micronization	tempered overnight moisture 24/100, heated at 90 °C with 115V infrared for 2.5 min	80	78	IVPD	
Chickpea	Boiling	90 min	84	89	IVPD	[112]
	Autoclaving	35 min at 15 lb pressure and 121 °C	84	90	IVPD	
	Microwave	15 min at 2450 MHz and dried at 50 °C for 20 h	84	89	IVPD	
Bambara groundnut	Soaking	overnight at room temp	79	76	IVPD	[109]
	Boiling	soaked and boiled for 120 min	79	49	IVPD	
	Boiling	unsoaked and boiled for 120 min	79	52	IVPD	
	Boiling	unsoaked and boiled in 2% NaCl for 120 min	79	51	IVPD	
	Roasting	roasted at 230 °C until colour change	79	42	IVPD	
Sorghum grain flour	Boiling	flour 1:10 (*w*/*v*) in water cooked in water bath for 20 min	53	30	IVPD	[110]
	Dry heating	90 min	53	50	IVPD	
	Popping	popped in hot-air oven and ground to powder	53	54	IVPD	
Pistachio	Roasting	115 °C for 30 min	94	85.19	SID (pig)	[74]
Soybean (ground)	Wet heating	80 °C for 1 min	46	52	SID (pig)	[108]
	Wet heating	100 °C for 6 min	46	73	SID (pig)	
	Wet heating	100 °C for 16 min	46	80	SID (pig)	
	Autoclaving	110 °C for 15 min	46	82	SID (pig)	
	Autoclaving	110 °C for 30 min	46	83	SID (pig)	
	Autoclaving	110 °C for 45 min	46	84	SID (pig)	
	Autoclaving	110 °C for 60 min	46	82	SID (pig)	
Soybean dried (whole)	Roasting	110–115 °C	53	72	SID (pig)	[113]
Red sorghum	Extrusion	Extruded at 182 °C and 14% moisture	53	70	IVPD	[114]

IVPD: In vitro protein digestibility [115]; SID: Standardized ileal digestibility [116]; TD: True protein digestibility (using isotope method) [107].

## References

[1] Afshin A., Sur P.J., Fay K.A., Cornaby L., Ferrara G., Salama J.S., Mullany E.C., Abate K.H., Abbafati C., Abebe Z. (2019). Health effects of dietary risks i n 195 countries, 1990–2017: A systematic analysis for the Global Burden of Disease Study 2017. Lancet.

[2] (2003). World Health Organization Diet, Nutrition, and the Prevention of Chronic Diseases: Report of a Joint WHO/FAO Expert Consultation.

[3] Herforth A., Arimond M., Álvarez-Sánchez C., Coates J., Christianson K., Muehlhoff E. (2019). a global review of food-based dietary guidelines. Adv. Nutr..

[4] Brown K.A., Timotijevic L., Barnett J., Shepherd R., Lähteenmäki L., Raats M.M. (2011). A review of consumer awareness, understanding and use of food-based dietary guidelines. Br. J. Nutr..

[5] Springmann M., Spajic L., Clark M.A., Poore J., Herforth A., Webb P., Rayner M., Scarborough P. (2020). The healthiness and sustainability of national and global food based dietary guidelines: Modelling study. BMJ.

[6] van de Kamp M.E., van Dooren C., Hollander A., Geurts M., Brink E.J., van Rossum C., Biesbroek S., de Valk E., Toxopeus I.B., Temme E.H.M. (2018). Healthy diets with reduced environmental impact?—The greenhouse gas emissions of various diets adhering to the Dutch food based dietary guidelines. Food Res. Int..

[7] The Eat-Lancet Commission, EAT-Lancet Commission Healthy Diets from Planet (2019). Food Planet Health.

[8] Fehér A., Gazdecki M., Véha M., Szakály M., Szakály Z. (2020). A comprehensive review of the benefits of and the barriers to the switch to a plant-based diet. Sustainability.

[9] Lassen A.D., Christensen L.M., Trolle E. (2020). Development of a Danish adapted healthy plant-based diet Based on the EAT-Lancet reference diet. Nutrients.

[10] Springmann M., Clark M., Mason-D’Croz D., Wiebe K., Bodirsky B.L., Lassaletta L., Vries W., Vermeulen S.J., Herrero M., Carlson K.M. (2018). Options for keeping the food system within environmental limits. Nature.

[11] Searchinger T., Hanson C., Ranganathan J., Lipinski B., Waite R., Winterbottom R., Dinshaw A., Heimlich R., Boval M., Chemineau P. (2014). Creating a Sustainable Food Future. A Menu of Solutions to Sustainably Feed More Than 9 Billion People by 2050. World Resources Report 2013–14: Interim Findings.

[12] Selinger E., Kühn T., Procházková M., Anděl M., Gojda J. (2019). Vitamin B12 deficiency is prevalent among Czech vegans who do not use Vitamin B12 supplements. Nutrients.

[13] Groufh-Jacobsen S., Hess S.Y., Aakre I., Folven Gjengedal E.L., Blandhoel Pettersen K., Henjum S. (2020). Vegans, vegetarians and pescatarians are at risk of iodine deficiency in Norway. Nutrients.

[14] Weikert C., Trefflich I., Menzel J., Obeid R., Longree A., Dierkes J., Meyer K., Herter-Aeberli I., Mai K., Stangl G.I. (2020). Vitamin and mineral status in a vegan diet. Dtsch. Arztebl. Int..

[15] Bakaloudi D.R., Halloran A., Rippin H.L., Oikonomidou A.C., Dardavesis T.I., Williams J., Wickramasinghe K., Breda J., Chourdakis M. (2021). Intake and adequacy of the vegan diet. A systematic review of the evidence. Clin. Nutr..

[16] Hansen T.H., Madsen M.T.B., Jørgensen N.R., Cohen A.S., Hansen T., Vestergaard H., Pedersen O., Allin K.H. (2018). Bone turnover, calcium homeostasis, and vitamin D status in Danish vegans. Eur. J. Clin. Nutr..

[17] Weaver C.M., Plawecki K.L. (1994). Dietary calcium: Adequacy of a vegetarian diet. Am. J. Clin. Nutr..

[18] Saunders A.V., Craig W.J., Baines S.K. (2013). Zinc and vegetarian diets. Med. J. Aust..

[19] Hunt J.R. (2003). Bioavailability of iron, zinc, and other trace minerals from vegetarian diets. Am. J. Clin. Nutr..

[20] Wolfe R.R., Baum J.I., Starck C., Moughan P.J. (2018). Factors contributing to the selection of dietary protein food sources. Clin. Nutr..

[21] Moughan P.J. (2021). Population protein intakes and food sustainability indices: The metrics matter. Glob. Food Sec..

[22] Moughan P.J., Wolfe R.R. (2019). Determination of dietary amino acid digestibility in humans. J. Nutr..

[23] Herreman L., Nommensen P., Pennings B., Laus M.C. (2020). Comprehensive overview of the quality of plant- And animal-sourced proteins based on the digestible indispensable amino acid score. Food Sci. Nutr..

[24] Ertl P., Knaus W., Zollitsch W. (2016). An approach to including protein quality when assessing the net contribution of livestock to human food supply. Animal.

[25] Berardy A., Johnston C.S., Plukis A., Vizcaino M., Wharton C. (2019). Integrating protein quality and quantity with environmental impacts in life cycle assessment. Sustainability.

[26] EFSA Panel on Dietetic Products and Allergies (NDA) N. (2010). Scientific Opinion on Dietary Reference Values for fats, including saturated fatty acids, polyunsaturated fatty acids, monounsaturated fatty acids, trans fatty acids, and cholesterol. EFSA J..

[27] EFSA Panel on Dietetic Products, Nutrition and Allergies (NDA) (2012). Scientific Opinion on Dietary Reference Values for protein. EFSA J..

[28] Boye J., Wijesinha-Bettoni R., Burlingame B. (2012). Protein quality evaluation twenty years after the introduction of the protein digestibility corrected amino acid score method. Br. J. Nutr..

[29] Wu G. (2009). Amino acids: Metabolism, functions, and nutrition. Amino Acids.

[30] (2013). FAO Dietary Protein Quality Evaluation in Human Nutrition: Report of an FAO Expert Consultation.

[31] (2005). IoM (Institute of Medicine) Dietary Reference Intakes for Energy, Carbohydrate, Fiber, Fat, Fatty Acids, Cholesterol, Protein, and Amino Acids (Macronutrients).

[32] (2007). Joint WHO/FAO/UNU Expert Consultation Protein and Amino Acid Requirements in Human Nutrition.

[33] Gilani G.S., Lee N., Caballero B. (2003). PROTEIN|Quality. Encyclopedia of Food Sciences and Nutrition.

[34] Institute of Medicine (U.S.) Food and Nutrition Board (1998). Dietary Reference Intakes: A Risk Assessment Model for Establishing Upper Intake Levels for Nutrients.

[35] Wang D., Ye J., Shi R., Zhao B., Liu Z., Lin W., Liu X. (2022). Dietary protein and amino acid restriction: Roles in metabolic health and aging-related diseases. Free Radic. Biol. Med..

[36] Baum J., Børsheim E., Allman B., Walker S. (2020). Health benefits of dietary protein throughout the life cycle. The Health Benefits of Foods—Current Knowledge and Further Development.

[37] Millward D.J. (2012). Identifying recommended dietary allowances for protein and amino acids: A critique of the 2007 WHO/FAO/UNU report. Br. J. Nutr..

[38] Pillai R.R., Kurpad A.V. (2012). Amino acid requirements in children and the elderly population. Br. J. Nutr..

[39] Bröer S., Fairweather S.J. (2018). Amino acid transport across the mammalian intestine. Compr. Physiol..

[40] Trommelen J., Tomé D., van Loon L.J.C. (2021). Gut amino acid absorption in humans: Concepts and relevance for postprandial metabolism. Clin. Nutr. Open Sci..

[41] Singh H., Gallier S., Boland M., Golding M. (2014). Chapter 2—Processing of Food Structures in the Gastrointestinal Tract and Physiological Responses. Singh Digestion and Health.

[42] Bornhorst G.M., Paul Singh R. (2014). Gastric digestion in vivo and in vitro: How the structural aspects of food influence the digestion process. Annu. Rev. Food Sci. Technol..

[43] Protein Digestion, Absorption and Metabolism. https://med.libretexts.org/@go/page/1869.

[44] Moran E.T. (2016). Gastric digestion of protein through pancreozyme action optimizes intestinal forms for absorption, mucin formation and villus integrity. Anim. Feed Sci. Technol..

[45] Jahan-Mihan A., Luhovyy B.L., Khoury D., Harvey Anderson G. (2011). Dietary proteins as determinants of metabolic and physiologic functions of the gastrointestinal tract. Nutrients.

[46] Heda R., Toro F., Tombazzi C.R. (2021). Physiology, Pepsin.

[47] van der Wielen N., de Vries S., Gerrits W., Jannink K., Moughan P., Mensink M., Hendriks W. (2021). Presence of unabsorbed free amino acids at the end of the small intestine of humans and pigs: Potential implications for amino acid bioavailability. Curr. Dev. Nutr..

[48] van der Wielen N., Moughan P.J., Mensink M. (2017). Amino acid absorption in the large intestine of humans and porcine models. J. Nutr..

[49] Neis E.P.J.G., Dejong C.H.C., Rensen S.S. (2015). The role of microbial amino acid metabolism in host metabolism. Nutrients.

[50] Wolfe R.R., Rutherfurd S.M., Kim I.Y., Moughan P.J. (2016). Protein quality as determined by the digestible indispensable amino acid score: Evaluation of factors underlying the calculation. Nutr. Rev..

[51] FAO/WHO Protein Quality Evaluation (1991). Report of the Joint FAO/WHO Expert Consultation.

[52] Hoffman J.R., Falvo M.J. (2004). Protein—Which is best?. J. Sport. Sci. Med..

[53] Kurpad A.V., Caballero B. (2013). Protein: Quality and Sources.

[54] Millward D.J., Jackson A.A. (2004). Protein/energy ratios of current diets in developed and developing countries compared with a safe protein/energy ratio: Implications for recommended protein and amino acid intakes. Public Health Nutr..

[55] Sá A.G.A., Moreno Y.M.F., Carciofi B.A.M. (2019). Food processing for the improvement of plant proteins digestibility. Crit. Rev. Food Sci. Nutr..

[56] Gilani S., Tomé D., Moughan P., Burlingame B. (2012). Report of a Sub-Committee of the 2011 FAO Consultation on “Protein Quality Evaluation in Human Nutrition” on: The assessment of amino acid digestibility in foods for humans and including a collation of published ileal amino acid digestibility data for. FAO Expert.

[57] Mathai J.K., Liu Y., Stein H.H. (2017). Values for digestible indispensable amino acid scores (DIAAS) for some dairy and plant proteins may better describe protein quality than values calculated using the concept for protein digestibility-corrected amino acid scores (PDCAAS). Br. J. Nutr..

[58] FAO/WHO/UNU (1985). Energy and Protein Requirements: Report of a Joint FAO/WHO/UNU Expert Consultation.

[59] Schaafsma G. (2000). The protein digestibility–Corrected amino acid score. J. Nutr..

[60] Marinangeli C.P.F., House J.D. (2017). Potential impact of the digestible indispensable amino acid score as a measure of protein quality on dietary regulations and health. Nutr. Rev..

[61] Devi S., Varkey A., Sheshshayee M.S., Preston T., Kurpad A.V. (2018). Measurement of protein digestibility in humans by a dual-tracer method. Am. J. Clin. Nutr..

[62] van der Wielen N., Khodorova N.V., Gerrits W.J.J., Gaudichon C., Calvez J., Tomé D., Mensink M. (2020). Blood 15N:13C enrichment ratios are proportional to the ingested quantity of protein with the dual-tracer approach for determining amino acid bioavailability in humans. J. Nutr..

[63] Food and Agriculture Organization(FAO) (2017). Protein Quality Assessment in Follow-Up Formula for Young Children and Ready to Use Therapeutic Foods: Report of the FAO Expert Working Group.

[64] Guillin F.M., Gaudichon C., Guérin-Deremaux L., Lefranc-Millot C., Airinei G., Khodorova N., Benamouzig R., Pomport P.-H., Martin J., Calvez J. (2021). Real ileal amino acid digestibility of pea protein compared to casein in healthy humans: A randomized trial. Am. J. Clin. Nutr..

[65] Calvez J., Benoit S., Piedcoq J., Khodorova N., Azzout-Marniche D., Tomé D., Benamouzig R., Airinei G., Gaudichon C. (2021). Very low ileal nitrogen and amino acid digestibility of zein compared to whey protein isolate in healthy volunteers. Am. J. Clin. Nutr..

[66] Deglaire A., Bos C., Tomé D., Moughan P.J. (2009). Ileal digestibility of dietary protein in the growing pig and adult human. Br. J. Nutr..

[67] Gaudichon C., Calvez J. (2021). Determinants of amino acid bioavailability from ingested protein in relation to gut health. Curr. Opin. Clin. Nutr. Metab. Care.

[68] Lee W.T.K., Weisell R., Albert J., Tomé D., Kurpad A.V., Uauy R. (2016). Research approaches and methods for evaluating the protein quality of human foods proposed by an fao expert working group in 2014. J. Nutr..

[69] Deglaire A., Moughan P.J. (2012). Animal models for determining amino acid digestibility in humans—A review. Br. J. Nutr..

[70] Rowan A.M., Moughan P.J., Wilson M.N., Maher K., Tasman-Jones C. (1994). Comparison of the ileal and faecal digestibility of dietary amino acids in adult humans and evaluation of the pig as a model animal for digestion studies in man. Br. J. Nutr..

[71] Hendriks W.H., Van Baal J., Bosch G. (2012). Ileal and faecal protein digestibility measurement in humans and other non-ruminants—A comparative species view. Br. J. Nutr..

[72] Rutherfurd S.M., Fanning A.C., Miller B.J., Moughan P.J. (2015). Protein digestibility-corrected amino acid scores and digestible indispensable amino acid scores differentially describe protein quality in growing male rats. J. Nutr..

[73] Nitrayová S., Brestenský M., Patráš P. (2018). Comparison of two methods of protein quality evaluation in rice, rye and barley as food protein sources in human nutrition. Potravin. Slovak J. Food Sci..

[74] Bailey H.M., Stein H.H. (2020). Raw and roasted pistachio nuts (*Pistacia vera* L.) are ‘good’ sources of protein based on their digestible indispensable amino acid score as determined in pigs. J. Sci. Food Agric..

[75] Rutherfurd S.M., Bains K., Moughan P.J. (2012). Available lysine and digestible amino acid contents of proteinaceous foods of India. Br. J. Nutr..

[76] Bailey H.M., Mathai J.K., Berg E.P., Stein H.H. (2020). Most meat products have digestible indispensable amino acid scores that are greater than 100, but processing may increase or reduce protein quality. Br. J. Nutr..

[77] Fanelli N.S., Bailey H.M., Guardiola L.V., Stein H.H. (2021). Values for digestible indispensable amino acid score (DIAAS) determined in pigs are greater for milk than for breakfast cereals, but DIAAS values for individual ingredients are additive in combined meals. J. Nutr..

[78] Hodgkinson S.M., Montoya C.A., Scholten P.T., Rutherfurd S.M., Moughan P.J. (2018). Cooking conditions affect the true ileal digestible amino acid content and digestible indispensable amino acid score (DIAAS) of bovine meat as determined in pigs. J. Nutr..

[79] Bailey H.M. (2018). Digestible Indispensable Amino Acid Scores for Meat Products. Master’s Thesis.

[80] Cervantes-Pahm S.K., Liu Y., Stein H.H. (2014). Digestible indispensable amino acid score and digestible amino acids in eight cereal grains. Br. J. Nutr..

[81] Han F., Han F., Wang Y., Fan L., Song G., Chen X., Jiang P., Miao H., Han Y. (2019). Digestible indispensable amino acid scores of nine cooked cereal grains. Br. J. Nutr..

[82] Guerra A., Etienne-Mesmin L., Livrelli V., Denis S., Blanquet-Diot S., Alric M. (2012). Relevance and challenges in modeling human gastric and small intestinal digestion. Trends Biotechnol..

[83] Nosworthy M.G., Franczyk A., Zimoch-Korzycka A., Appah P., Utioh A., Neufeld J., House J.D. (2017). Impact of processing on the protein quality of pinto bean (*Phaseolus vulgaris*) and buckwheat (*Fagopyrum esculentum Moench*) flours and blends, as determined by in vitro and in vivo methodologies. J. Agric. Food Chem..

[84] Bohn T., Carriere F., Day L., Deglaire A., Egger L., Freitas D., Golding M., Le Feunteun S., Macierzanka A., Menard O. (2018). Correlation between in vitro and in vivo data on food digestion. What can we predict with static in vitro digestion models?. Crit. Rev. Food Sci. Nutr..

[85] Rieder A., Afseth N.K., Böcker U., Knutsen S.H., Kirkhus B., Mæhre H.K., Ballance S., Wubshet S.G. (2021). Improved estimation of in vitro protein digestibility of different foods using size exclusion chromatography. Food Chem..

[86] Heo J.M., Kiarie E., Kahindi R.K., Maiti P., Woyengo T.A., Nyachoti C.M. (2012). Standardized ileal amino acid digestibility in egg from hyperimmunized hens fed to weaned pigs. J. Anim. Sci..

[87] Han F., Moughan P.J., Li J., Pang S. (2020). Digestible indispensable amino acid scores (DIAAS) of six cooked CHINESE pulses. Nutrients.

[88] Abelilla J.J., Liu Y., Stein H.H. (2018). Digestible indispensable amino acid score (DIAAS) and protein digestibility corrected amino acid score (PDCAAS) in oat protein concentrate measured in 20- to 30-kilogram pigs. J. Sci. Food Agric..

[89] Bailey H.M., Stein H.H. (2019). Can the digestible indispensable amino acid score methodology decrease protein malnutrition. Anim. Front..

[90] Mansilla W.D., Marinangeli C.P.F., Cargo-Froom C., Franczyk A., House J.D., Elango R., Columbus D.A., Kiarie E., Rogers M., Shoveller A.K. (2020). Comparison of methodologies used to define the protein quality of human foods and support regulatory claims. Appl. Physiol. Nutr. Metab..

[91] Grandison A.S. (2011). Postharvest handling and preparation of foods for processing. Food Process. Handb..

[92] Aluko R.E. (2018). 15—Food protein-derived peptides: Production, isolation, and purification. Proteins in Food Processing.

[93] Fabbri A.D.T., Crosby G.A. (2016). A review of the impact of preparation and cooking on the nutritional quality of vegetables and legumes. Int. J. Gastron. Food Sci..

[94] Nosworthy M.G., Medina G., Franczyk A.J., Neufeld J., Appah P., Utioh A., Frohlich P., House J.D. (2018). Effect of processing on the in vitro and in vivo protein quality of beans (*Phaseolus vulgaris* and *Vicia Faba*). Nutrients.

[95] Nosworthy M.G., Medina G., Franczyk A.J., Neufeld J., Appah P., Utioh A., Frohlich P., Tar’an B., House J.D. (2020). Thermal processing methods differentially affect the protein quality of Chickpea (*Cicer arietinum*). Food Sci. Nutr..

[96] Hurrell R.F., Finot P.A. (1983). Food processing and storage as a determinant of protein and amino acid availability. Experientia Suppl..

[97] Rérat A., Calmes R., Vaissade P., Finot P.-A.A. (2002). Nutritional and metabolic consequences of the early Maillard reaction of heat treated milk in the pig. Significance for man. Eur. J. Nutr..

[98] Salazar-Villanea S., Butré C.I., Wierenga P.A., Bruininx E.M.A.M.A.M., Gruppen H., Hendriks W.H., van der Poel A.F.B.B. (2018). Apparent ileal digestibility of Maillard reaction products in growing pigs. PLoS ONE.

[99] Nyakayiru J., van Lieshout G.A.A., Trommelen J., van Kranenburg J., Verdijk L.B., Bragt M.C.E., van Loon L.J.C. (2020). The glycation level of milk protein strongly modulates post-prandial lysine availability in humans. Br. J. Nutr..

[100] Zenker H.E., Van Lieshout G.A.A., Van Gool M.P., Bragt M.C.E., Hettinga K.A. (2020). Lysine blockage of milk proteins in infant formula impairs overall protein digestibility and peptide release. Food Funct..

[101] Rutherfurd S.M., Moughan P.J. (2005). Digestible reactive lysine in selected milk-based products. J. Dairy Sci..

[102] Torbatinejad N.M., Rutherfurd S.M., Moughan P.J. (2005). Total and reactive lysine contents in selected cereal-based food products. J. Agric. Food Chem..

[103] Erbersdobler H.F., Hupe A. (1991). Determination of lysine damage and calculation of lysine bio-availability in several processed foods. Z. ErnAhrungswiss..

[104] Gilani G.S., Xiao C.W., Cockell K.A. (2012). Impact of antinutritional factors in food proteins on the digestibility of protein and the bioavailability of amino acids and on protein quality. Br. J. Nutr..

[105] Friedman M. (2004). Nutrition|Effects of food processing. Encycl. Grain Sci..

[106] Friedman M. (1999). Chemistry, biochemistry, nutrition, and microbiology of lysinoalanine, lanthionine, and histidinoalanine in food and other proteins. J. Agric. Food Chem..

[107] Evenepoel P., Geypens B., Luypaerts A., Hiele M., Ghoos Y., Rutgeerts P. (1998). Digestibility of cooked and raw egg protein in humans as assessed by stable isotope techniques. J. Nutr..

[108] Kaewtapee C., Eklund M., Wiltafsky M., Piepho H.-P.P., Mosenthin R., Rosenfelder P. (2017). Influence of wet heating and autoclaving on chemical composition and standardized ileal crude protein and amino acid digestibility in full-fat soybeans for pigs1,2. J. Anim. Sci..

[109] El-gasim A.Y.A., Abdalla A.A. (2007). Effect of domestic processing methods on chemical composition, in vitro digestibility of protein and starch and functional properties of Bambara groundnut (*Voandzeia subterranea*) Seed. Res. J. Agric. Biol. Sci..

[110] Correia I., Nunes A., Barros A.S., Delgadillo I. (2010). Comparison of the effects induced by different processing methods on sorghum proteins. J. Cereal Sci..

[111] Khattab R.Y., Arntfield S.D., Nyachoti C.M. (2009). Nutritional quality of legume seeds as affected by some physical treatments, Part 1: Protein quality evaluation. LWT Food Sci. Technol..

[112] Alajaji S.A., El-Adawy T.A. (2006). Nutritional composition of chickpea (*Cicer arietinum* L.) as affected by microwave cooking and other traditional cooking methods. J. Food Compos. Anal..

[113] Kaewtapee C., Mosenthin R., Nenning S., Wiltafsky M., Schäffler M., Eklund M., Rosenfelder-Kuon P. (2018). Standardized ileal digestibility of amino acids in European soya bean and rapeseed products fed to growing pigs. J. Anim. Physiol. Anim. Nutr..

[114] Llopart E.E., Drago S.R., De Greef D.M., Torres R.L., González R.J. (2014). Effects of extrusion conditions on physical and nutritional properties of extruded whole grain red sorghum (*Sorghum* spp.). Int. J. Food Sci. Nutr..

[115] Salgó A., Ganzler K., Jécsai J. (1985). Simple enzymic methods for prediction of plant protein digestibility. Amin. Acid Compos. Biol. Value Cereal Proteins.

[116] Stein H.H., Fuller M.F., Moughan P.J., Sève B., Mosenthin R., Jansman A.J.M., Fernández J.A., de Lange C.F.M. (2007). Definition of apparent, true, and standardized ileal digestibility of amino acids in pigs. Livest. Sci..

[117] Salazar-Villanea S., Hendriks W.H., Bruininx E.M.A.M., Gruppen H., van der Poel A.F.B. (2016). Protein structural changes during processing of vegetable feed ingredients used in swine diets: Implications for nutritional value. Nutr. Res. Rev..

[118] Samtiya M., Aluko R.E., Dhewa T. (2020). Plant food anti-nutritional factors and their reduction strategies: An overview. Food Prod. Process. Nutr..

[119] Avilés-Gaxiola S., Chuck-Hernández C., Serna Saldívar S.O. (2018). Inactivation methods of trypsin inhibitor in legumes: A review. J. Food Sci..

[120] Alonso R., Aguirre A., Marzo F. (2000). Effects of extrusion and traditional processing methods on antinutrients and in vitro digestibility of protein and starch in Faba and kidney beans. Food Chem..

[121] van Lieshout G.A.A.A., Lambers T.T., Bragt M.C.E.E., Hettinga K.A. (2019). How processing may affect milk protein digestion and overall physiological outcomes: A systematic review. Crit. Rev. Food Sci. Nutr..

[122] Dias D.R., Abreu C.M.P.D., Silvestre M.P.C., Schwan R.F. (2010). In vitro protein digestibility of enzymatically pre-treated bean (*Phaseolus vulgaris* L.) flour using commercial protease and Bacillus sp. protease. Food Sci. Technol..

[123] Joye I. (2019). Protein digestibility of cereal products. Foods.

[124] Fastinger N.D., Mahan D.C. (2003). Effect of soybean meal particle size on amino acid andenergy digestibility in grower-finisher swine1234. J. Anim. Sci..

[125] Kim J.C., Mullan B.P., Heo J.M., Hansen C.F., Pluske J.R. (2009). Decreasing dietary particle size of lupins increases apparent ileal amino acid digestibility and alters fermentation characteristics in the gastrointestinal tract of pigs. Br. J. Nutr..

[126] Pennings B., Groen B.B.L., van Dijk J.-W., de Lange A., Kiskini A., Kuklinski M., Senden J.M.G., van Loon L.J.C. (2013). Minced beef is more rapidly digested and absorbed than beef steak, resulting in greater postprandial protein retention in older men. Am. J. Clin. Nutr..

[127] Aoyama S., Kim H.-K., Hirooka R., Tanaka M., Shimoda T., Chijiki H., Kojima S., Sasaki K., Takahashi K., Makino S. (2021). Distribution of dietary protein intake in daily meals influences skeletal muscle hypertrophy via the muscle clock. Cell Rep..

[128] Engelking L.R. (2015). Chapter 44—Vitamin A. Textbook of Veterinary Physiological Chemistry.

[129] Atherton P.J., Etheridge T., Watt P.W., Wilkinson D., Selby A., Rankin D., Smith K., Rennie M.J. (2010). Muscle full effect after oral protein: Time-dependent concordance and discordance between human muscle protein synthesis and mTORC1 signaling. Am. J. Clin. Nutr..

[130] Groen B.B.L., Horstman A.M., Hamer H.M., de Haan M., van Kranenburg J., Bierau J., Poeze M., Wodzig W.K.W.H., Rasmussen B.B., van Loon L.J.C. (2015). Post-prandial protein handling: You are what you just ate. PLoS ONE.

[131] Dangin M., Boirie Y., Garcia-Rodenas C., Gachon P., Fauquant J., Callier P., Ballèvre O., Beaufrère B. (2001). The digestion rate of protein is an independent regulating factor of postprandial protein retention. Am. J. Physiol. Metab..

[132] Areta J.L., Burke L.F., Ross M.L., Camera D.M., West D.W.D., Broad E.M., Jeacocke N.A., Moore D.R., Stellingwerff T., Phillips S.M. (2013). Timing and distribution of protein ingestion during prolonged recovery from resistance exercise alters myofibrillar protein synthesis. J. Physiol..

[133] Witard O.C., Jackman S.R., Breen L., Smith K., Selby A., Tipton K.D. (2014). Myofibrillar muscle protein synthesis rates subsequent to a meal in response to increasing doses of whey protein at rest and after resistance exercise. Am. J. Clin. Nutr..

[134] Giordano M., Castellino P., DeFronzo R.A. (1996). Differential responsiveness of protein synthesis and degradation to amino acid availability in humans. Diabetes.

[135] Luiking Y.C., Deutz N.E.P., Jäkel M., Soeters P.B. (2005). Casein and soy protein meals differentially affect whole-body and splanchnic protein metabolism in healthy humans. J. Nutr..

[136] Schutz Y. (2011). Protein turnover, ureagenesis and gluconeogenesis. Int. J. Vitam. Nutr. Res..

[137] Ishikawa-Takata K., Takimoto H. (2018). Current protein and amino acid intakes among Japanese people: Analysis of the 2012 national health and nutrition survey. Geriatr. Gerontol. Int..

[138] USDA What We Eat in America Database 2017–2018. https://www.ars.usda.gov/northeast-area/beltsville-md-bhnrc/beltsville-human-nutrition-research-center/food-surveys-research-group/docs/wweia-data-tables/.

[139] Tieland M., Borgonjen-Van den Berg K.J., Van Loon L.J.C., De Groot L.C.P.G.M. (2015). Dietary protein intake in dutch elderly people: A focus on protein sources. Nutrients.

[140] Mamerow M.M., Mettler J.A., English K.L., Casperson S.L., Arentson-Lantz E., Sheffield-Moore M., Layman D.K., Paddon-Jones D. (2014). Dietary protein distribution positively influences 24-h muscle protein synthesis in healthy adults. J. Nutr..

[141] Schoenfeld B.J., Aragon A.A. (2018). How much protein can the body use in a single meal for muscle-building?. J. Int. Soc. Sports Nutr..

[142] van den Borne J.J.G.C., Alferink S.J.J., Heetkamp M.J.W., Jacobs A.A.A., Verstegen M.W.A., Gerrits W.J.J. (2012). Asynchronous supply of indispensable amino acids reduces protein deposition in milk-fed calves. J. Nutr..

[143] Hou Y., Yin Y., Wu G. (2015). Dietary essentiality of ‘nutritionally non-essential amino acids’ for animals and humans. Exp. Biol. Med..

[144] Han F., Moughan P.J., Li J., Stroebinger N., Pang S. (2021). The complementarity of amino acids in cooked pulse/cereal blends and effects on DIAAS. Plants.

[145] Pencharz P.B., Elango R., Wolfe R.R. (2016). Recent developments in understanding protein needs—How much and what kind should we eat?. Appl. Physiol. Nutr. Metab..

[146] Riddet Institute DELTA Model 1.3 Web. https://sustainablenutritioninitiative.com/the-delta-model/explore-the-future/.

